# Hierarchical Assembly of Carbon Dots with Full‐Solar‐Spectrum Absorption for Solar Energy Applications

**DOI:** 10.1002/advs.202417457

**Published:** 2025-03-17

**Authors:** Lei Li, Di Li, Yanfei Qu, Ruoyu Zhang, Shuo Qi, Mengyao Liu, Haohao Bi, Tao Jia, Songnan Qu, Weitao Zheng

**Affiliations:** ^1^ Key Laboratory of Automobile Materials College of Materials Science and Engineering Jilin University Changchun 130012 P. R. China; ^2^ Key Laboratory of Forest Plant Ecology Ministry of Education Engineering Research Center of Forest Bio‐Preparation College of Chemistry Chemical Engineering and Resource Utilization Northeast Forestry University Harbin 150040 P. R. China; ^3^ Joint Key Laboratory of the Ministry of Education Institute of Applied Physics and Materials Engineering University of Macau Avenida da Universidade, Taipa Macau SAR 999078 P. R. China

**Keywords:** assembly, carbon dots, full‐solar‐spectrum absorption, hierarchical assembly, photothermal conversion

## Abstract

Carbon dots (CDs) featuring low‐cost, non‐toxic, and appealing optical properties demonstrate promising applications in energy, e.g. solar energy capture and conversion. However, it remains a significant challenge to expand the absorption bands of CDs from visible to near‐infrared (NIR) spectral regions to harness the entire spectrum of sunlight for efficient solar energy utilization. Herein, hierarchical assemblies of CDs (HA‐CDs) are constructed by stepwise assembling monodispersed ultraviolet‐absorbing CDs to water‐soluble visible‐NIR absorbing supra‐CDs (PA‐CDs), and then complexing PA‐CDs with Fe^3+^ ions to form 3D porous architectures (HA‐CDs) with full solar spectrum absorption and good water resistance. Notably, the HA‐CDs exhibit good hydrophilicity and superior photothermal conversion efficiency of 84% under simulated solar irradiation. The facile Fe^3+^ ion cross‐linking assembly property enables the in situ preparation of HA‐CDs on various fabric substrates, resulting in low‐cost, high‐performance photothermal conversion products. High‐performance 2D solar‐driven interfacial water evaporation, electricity generation, and water‐electricity cogeneration have been demonstrated in the HA‐CDs in situ coated fabric (HA‐CDs‐fabric). This study provides a novel and effective design approach for the development of high‐performance CD‐based photothermal materials for solar energy applications.

## Graphical abstract

Hierarchical assemblies of carbon dots are constructed, achieving full‐solar‐spectrum absorption, superior photothermal properties, and good hydrophilicity. These assemblies can be in situ prepared on a fabric, which exhibits good performance for applications in solar‐driven interfacial water evaporation and electricity generation. This paper provides a novel and practical design strategy for developing high‐performance carbon‐based photothermal materials for solar energy applications.

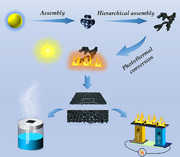

## 1 Introduction

To address the current issues of energy shortage and freshwater scarcity caused by human society's rapid development, solar‐heat conversion technology has received widespread attention because it can effectively capture and utilize sustainable solar energy at a low cost to achieve solar‐driven water evaporation and thermoelectric power generation.^[^ ^1^, ^2^ ^]^ To efficiently convert solar energy into heat, it is critical to explore photothermal materials with sufficient solar absorption and high photothermal conversion efficiency. To date, a variety of photothermal materials have been developed, including metallic nanoparticles, inorganic/organic semiconductors, carbon‐based materials, and their hybrid composites.^[^ ^3^, ^4^, ^5^ ^]^. Among these, carbon‐based materials stand out for their potential in solar energy capture and conversion, which is attributed to their abundant raw materials, non‐toxic nature, and excellent environmental friendliness.^[^ ^6^ ^]^ Nonetheless, the fabrication of carbon‐based materials often demands calcination at high temperatures (typically exceeding 400 °C) in an inert atmosphere, which is notably energy‐intensive.

Carbon dots (CDs) have emerged as a new family of 0D carbon‐based nanomaterials which typically exhibit a quasi‐spherical shape with diameters less than 10 nm, and have sp^2^/sp^3^ hybridized carbon cores surrounded by a variety of chemical functional groups. CDs are often synthesized through simple and scalable processes, starting from a variety of small molecules, natural materials, or bulk carbon sources, which result in low preparation costs, ease of structure modification, and well‐documented biocompatibility.^[^ ^7^, ^8^ ^]^ More importantly, CDs have appealing photophysical properties such as tunable optical bandgap, efficient photoluminescence, photothermal conversion, and good photostability. These advantages make CDs a rising star in the fields of energy, photoelectronics, and biomedicine.^[^ ^9^, ^10^, ^11^, ^12^, ^13^ ^]^ In recent years, CDs have demonstrated good performance in solar energy harvesting and photothermal conversion, highlighting their potential in applications such as solar‐driven interfacial water evaporation and thermoelectric generation.^[^ ^14^, ^15^, ^16^, ^17^ ^]^ Nevertheless, CDs used in solar energy harvesting are still rare, because the absorption bands of CDs are typically located in the visible region. Given that nearly half of the solar energy lies in the near‐infrared (NIR) region, it is of significant importance to expand the absorption range of CDs into the NIR region to fully capture the solar energy and enhance the efficiency of solar‐to‐heat conversion.

It is well established that strategies such as extending the π‐conjugation of the carbon‐based cores,^[^ ^18^, ^19^ ^]^ doping with heteroatoms,^[^ ^20^, ^21^, ^22^ ^]^ and surface modification^[^ ^23^, ^24^ ^]^ can broaden the absorption bands of CDs into longer wavelength regions, thereby improving their photothermal conversion capacities. However, achieving significant enhancement of absorption in the NIR region remains a challenge. In addition to these approaches, assembly has been demonstrated to be an effective pathway in regulating the energy structures of CDs due to the synergistic effects of intrinsic electron structures and interparticle interactions. Moreover, the abundant surface groups on CDs provide active sites for supramolecular interactions or chemical bonding, which can drive the formation of assemblies.^[^ ^25^, ^26^ ^]^ Liang et al. fused CDs into carbon nanorolls by interface dehydration, and the interlayer π−π conjugation in the compact rolling geometry of the assemblies contributed to their enhanced absorption band at 665 nm compared with the individual CDs.^[^ ^27^ ^]^ Hou et al. discovered that increasing the concentration of CD suspensions led to the formation of branch‐shaped CD assemblies, which resulted in enhanced absorbance throughout the UV–vis region with a notably redshifted absorption edge and hence good photothermal conversion capability.^[^ ^15^ ^]^

In our previous work, water‐soluble CD assemblies (PA‐CDs) with strong visible‐to‐NIR absorption bands and efficient NIR photothermal conversion were constructed by assembling UV‐absorbing CDs (F‐CDs) via hydrogen bonding and possible electrostatic interactions.^[^ ^28^ ^]^ The electron transitions within the coupled surface energy levels from adjacent F‐CDs in the assembly contributed to the emerged visible to NIR absorption. However, PA‐CDs formed through weak interparticle interactions were less stable (vide infra) in water, and their good water solubility became their drawback when used as photothermal evaporators, due to their insufficient water resistance. These problems hindered the applications of water‐soluble PA‐CDs in solar‐driven interfacial water evaporation. Herein, exploring the PA‐CDs with broadband absorption as building blocks, we have facilely developed the hierarchical assemblies of CDs (HA‐CDs) through the coordination interactions of PA‐CDs with Fe^3+^. The HA‐CDs possess water‐insoluble, cross‐linked porous nanostructures with hydrophilic groups on the surface, which improve the stability of the assemblies and endow the HA‐CDs‐based photothermal evaporator with water resistance and hydrophilicity. Most importantly, the HA‐CDs exhibit absorption bands covering the entire solar spectral region and exceptional photothermal conversion efficiency up to 84% under simulated solar irradiation. Due to the facile Fe^3+^ ion cross‐linking assembly property, a planar solar absorber based on the HA‐CDs in situ coated fabric (HA‐CDs‐fabric) has been developed. Ultimately, the 2D evaporator made from HA‐CDs‐fabric exhibits superior solar‐to‐vapor performance with an evaporation rate of 1.45 kg m^−2^ h^−1^ and evaporation efficiency of 99.1% for saline water under 1 kW m^−2^ simulated solar irradiation. Moreover, the HA‐CDs‐fabric solar absorber has been integrated into a photothermal‐electric conversion device, yielding a voltage of 225 mV, and the cogeneration of water and electricity has also been realized under 1 kW m^−2^ simulated solar irradiation.

## 2 Results and Discussion

### 2.1 Construction and Physicochemical Properties of HA‐CDs

**Figure 1:**
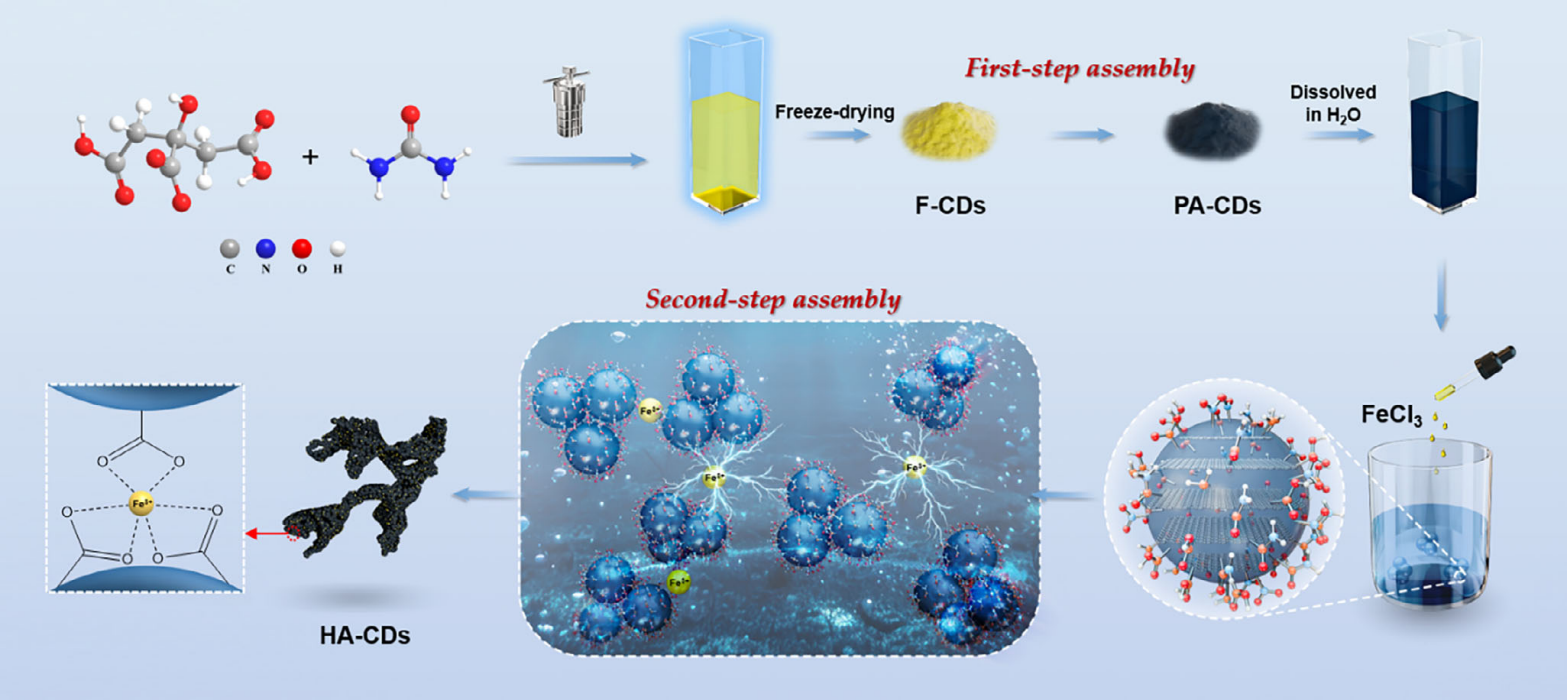
Schematic illustration of fabrication procedures of F‐CDs, PA‐CDs, and HA‐CDs, and the formation mechanism of the HA‐CD hierarchical assembly.

**Figure** **1** illustrates the design of HA‐CDs from the F‐CD building blocks, which are derived from the hydrothermal reaction of citric acid and urea. The preparation of HA‐CDs involves two steps of assembly. Initially, UV‐absorbing F‐CDs (λ_abs_ = 340 nm) were assembled into PA‐CDs. The PA‐CDs exhibited a strong visible to NIR absorption band (≈440−900 nm, Figure S4a, Supporting Information), together with quenching of fluorescence (Figure S4b, Supporting Information). A remarkable color change of the solution from the faint yellow of F‐CDs to the deep blue of PA‐CDs could be observed corresponding to the change in the absorption spectra (Figure S4a, Supporting Information). Subsequently, upon the addition of Fe^3+^ to the well‐dispersed aqueous solution of PA‐CDs, the second‐step assembly occurred and resulted in the formation of HA‐CDs precipitated from water. The reproducibility of HA‐CDs preparation was verified through parallel and scale‐up/down synthesis experiments (Figures S1 and S2 and Table S1, Supporting Information).

**Figure 2:**
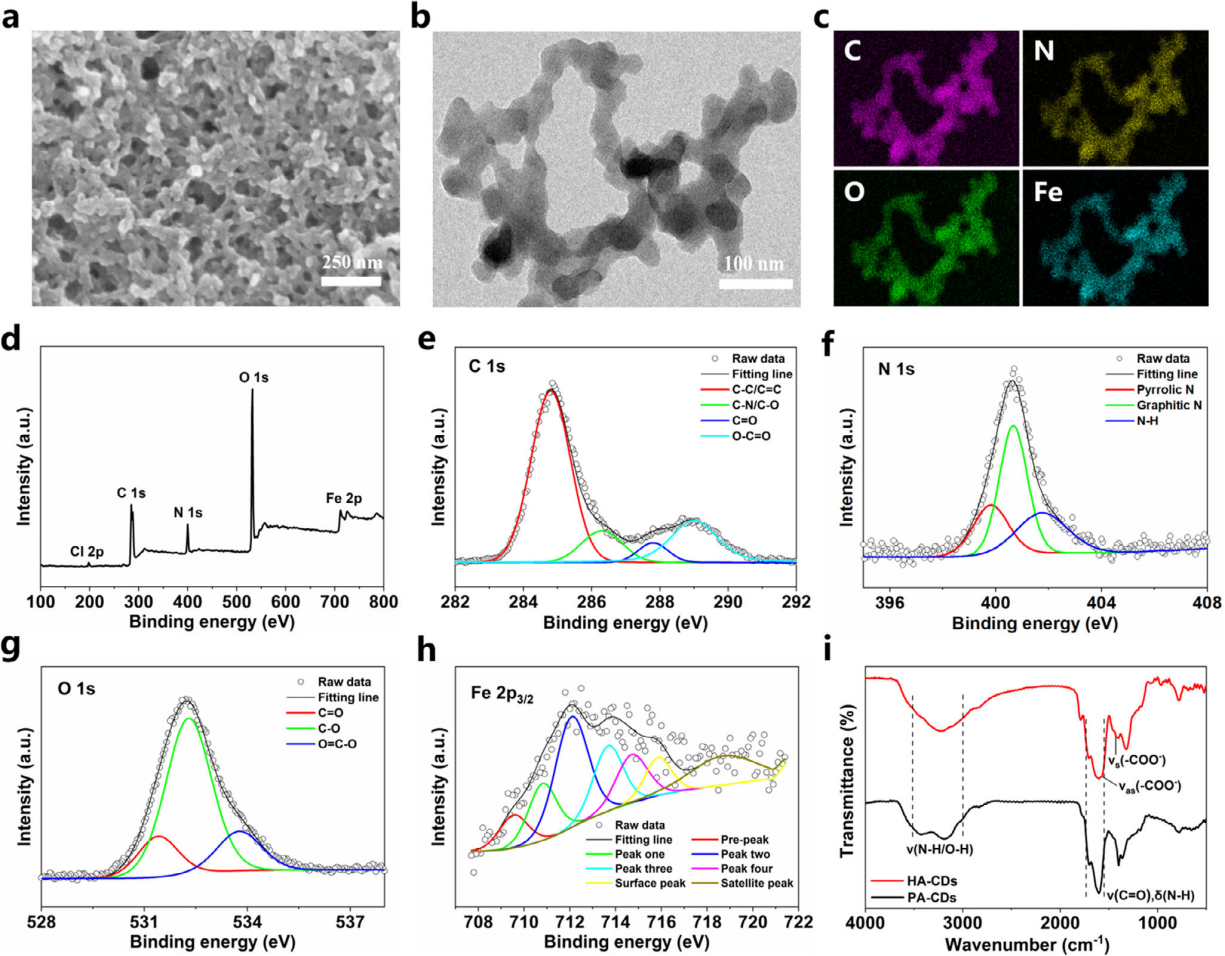
a) SEM image, b) TEM image, and c) EDX mapping of elemental distributions of HA‐CDs. d) XPS survey and e–h) high‐resolution C 1s, N 1s, O 1s, and Fe 2p XPS spectra of HA‐CDs. i) FTIR spectra of PA‐CDs and HA‐CDs.

The microstructures of HA‐CDs were investigated by scanning electron microscopy (SEM) and transmission electron microscopy (TEM). SEM image (**Figure** **2**a) disclosed that HA‐CDs were composed of crosslinked nanochains which established a 3D porous network architecture, and the porous structure was beneficial for harvesting solar energy.^[^ ^29^ ^]^ As shown in the TEM images (Figure S5b, Supporting Information), the PA‐CDs were monodispersed nanoparticles with relatively large sizes in the range of 20−30 nm as a result of the assembly of F‐CDs (with diameters in the range of 2−5 nm and lattice fringes with a spacing of 0.21 nm, Figure S5a, Supporting Information), consistent with our previous report.^[^ ^28^ ^]^ Following Fe^3+^‐triggered further assembly, the resulting interconnected string‐bead‐like morphology, with widths in the tens of nanometers, indicated that the nanochain was composed of Fe‐ion‐connected PA‐CD nanoparticles (Figure 2b). Additionally, the high‐resolution transmission electron microscope (HRTEM) image (Figure S5c, Supporting Information) revealed the presence of localized crystalline graphite phase with well‐defined lattice fringes (d = 0.21 nm corresponding to the (100) facet of graphitic carbon^[^ ^10^ ^]^) inside the assemblies, consistent with the lattice fringes in the starting F‐CD building blocks. The localized graphitization was also confirmed by the Raman spectrum of HA‐CDs (Figure S6, Supporting Information), which exhibited two broad peaks centered at ≈1350 and 1582 cm^−1^, corresponding to the D band and G band of the disordered and graphitized carbons, respectively. The relative intensity of the D band and G band (I_D_/I_G_ = 0.5) also indicated a relatively high carbon‐lattice‐structure content.^[^ ^30^ ^]^

The chemical compositions of HA‐CDs were further investigated using X‐ray photoelectron spectroscopy (XPS), TEM energy‐dispersive X‐ray spectroscopy (EDS), and Fourier transform infrared (FTIR) spectroscopy. The XPS survey spectrum and corresponding elemental analysis (Figure 2d) exhibited the presence of C (52.77 At%), N (9.66 At%), O (33.01%), Fe (3.56 At%) and Cl (1.01 At%) elements. The large decrease in the ratio of Cl to Fe in HA‐CDs as compared to FeCl_3_ indicated that Fe^3+^ coordinated with PA‐CDs by replacement of Cl. The TEM‐EDS elemental mapping (Figure 2c) confirmed a uniform distribution of the C, N, O, and Fe elements, revealing a homogeneous chemical composition throughout the hierarchical assemblies. In the high‐resolution XPS spectra (Figure 2e‒g), C 1s was detected as C−C/C═C (284.8 eV), C−N/C−O (286.3 eV), C═O (287.8 eV) and O−C═O (289 eV); N 1s was detected as pyrrolic N (399.8 eV), graphitic N (400.7 eV) and N−H (401.7 eV); and O 1s was detected as C═O (531.3 eV), C−O (532.2 eV) and O═C−O (533.8 eV), respectively.^[^ ^24^, ^31^ ^]^ Notably, the high‐resolution O 1s signal of PA‐CDs in the range of 532.4−536 eV, corresponding to the carboxyl groups (Figure S7a, Supporting Information), was obviously decreased after the formation of HA‐CDs (Figure S7b, Supporting Information), indicating the possible interactions between Fe^3+^ and carboxyl groups.^[^ ^32^ ^]^ Fe 2p_3/2_ (Figure 2h) could be fitted into seven peaks,^[^ ^33^ ^]^ in which the peaks centered at 709.5 and 715.7 eV belonged to the low‐BE pre‐peak and high‐BE surface peaks, respectively. Four strong peaks centered at 710.8, 712, 713.7, and 714.7 eV were attributed to the multiple splitting of Fe 2p and their satellite peak was at 718.9 eV. In the FTIR spectra (Figure 2i), the characteristic absorption bands of 𝑣(N−H)/𝑣(O−H) (3000−3500 cm^−1^) and 𝑣(C═O)/δ(N−H) (1550−1720 cm^−1^) occurred both in PA‐CDs and HA‐CDs, revealing the presence of carboxyl, hydroxyl, and amino groups in these two kinds of CD assemblies.^[^ ^34^ ^]^ For HA‐CDs, the FT‐IR spectra exhibited the 𝑣_as_(COO^−^) and 𝑣_s_(COO^−^) peaks of the carboxylate groups at 1572 and 1425 cm^−1^, respectively (Figure 2i; Figure S8, Supporting Information),^[^ ^35^ ^]^ indicating the carboxyl groups underwent coordination interactions with Fe^3+^.

Based on the structure and composition characterizations, we propose a possible formation mechanism of HA‐CDs (Figure 1). PA‐CDs are rich in carboxyl functional groups derived from the citric acid precursor and exhibit electronegativity with a zeta potential of −23.8 mV (Figure S9a, Supporting Information). These functional groups and Fe^3+^ can be attracted to each other via electrostatic interactions and form coordination bonds, which allows the further hierarchical assembly of PA‐CDs into HA‐CDs, as evidenced by the reduced zeta potential of −10.9 mV for HA‐CDs (Figure S9b, Supporting Information). In this process, Fe^3+^ acts as a “linker” to connect the adjacent PA‐CDs, resulting in the morphology of interconnected nanochains. In this regard, further assembly of PA‐CDs through coordination with Fe^3+^ can “strengthen” the assemblies and form porous precipitations, preventing water‐induced disassembly and achieving outstanding stability under a water‐immersed working environment.

HA‐CDs exhibited a broad absorption band covering the spectral range of 300−1800 nm from ultraviolet to NIR region, surpassing the absorption of PA‐CDs in the long wavelength region (Figure S10, Supporting Information). Moreover, it was found that the PA‐CDs were less stable in an aqueous solution,^[^ ^36^ ^]^ which could be attributed to the relatively weak interparticle interactions of hydrogen bonds and possible electrostatic interactions among the F‐CD building blocks.^[^ ^28^ ^]^ The characteristic visible to NIR absorption of PA‐CDs gradually weakened with time in water during 2‐days immersion (Figure S11a, Supporting Information). On the contrary, after being immersed in water for 2 days, the absorption spectra of HA‐CDs remained almost unchanged (Figure S11b, Supporting Information), demonstrating excellent antiwater stability. The stability of HA‐CDs was further confirmed in aqueous solutions with different pH values (pH 4.0, 6.86, and 9.18) and in a 10 wt% NaCl aqueous solution. Specifically, after soaking in these solutions for 12 h, the absorption spectra of HA‐CDs were essentially unchanged compared to that before immersion (Figure S12, Supporting Information).

On the basis of the broad absorption spectrum, the photothermal conversion capacity of HA‐CDs was evaluated by monitoring the temperature rise under 1 KW m^−2^ simulated solar irradiation. The temperature of the HA‐CD powders increased rapidly from 25 to 55 °C during the first minute and reached an equilibrium temperature of 59.5 °C at ≈2 min, and the temperature elevation of the HA‐CDs slightly surpassed that of the PA‐CDs with temperature increased from 25 to 55 °C under the same conditions (Figure S13a, Supporting Information). Upon three cycles of solar irradiation on and off, the temperature elevation curve of HA‐CD powders for each round was almost unchanged (Figure S13b, Supporting Information). When continuously exposed to 1 kW m^−2^ simulated solar irradiation for 2 h, the temperature of HA‐CD powders remained stable at ≈60 °C (Figure S13c, Supporting Information). This demonstrated good stability during the photothermal conversion process. Moreover, because of the effective absorption in the NIR region, HA‐CDs exhibit a photothermal response to the NIR laser irradiation. Under the exposure of a 1064 nm laser with power densities of 0.1, 0.2, and 0.3 W cm^−2^, HA‐CDs were able to reach equilibrium temperatures of 44.5, 58.5, and 72.5 °C, respectively, within 1 min and maintain these temperatures for an extended duration (Figure S14, Supporting Information). Furthermore, the photothermal conversion efficiency was measured to be 84% under simulated 1 kW m^−2^ solar irradiation (detailed calculation refers to Supporting Information), which clearly showed a competitive advantage over the reported photothermal materials.^[^ ^37^, ^38^, ^39^ ^]^

As reported in our previous work, the inhomogeneous surface‐confined charges evoked surface trap‐state energy levels on F‐CDs. Upon assembly into PA‐CDs, the interparticle electron transitions within the coupled surface energy levels of adjacent F‐CDs contributed to the enhanced red‐to‐NIR absorption and photothermal conversion capability.^[^ ^28^ ^]^ Following secondary assembly through coordination of PA‐CDs with Fe^3+^, stronger interparticle interactions were established in the resulting HA‐CDs, leading to tighter particle packing and enhanced assembly stability. This may further enhance the interparticle surface electron transitions, which is conducive to extending the absorption spectrum of HA‐CDs toward longer wavelengths and improving photothermal conversion. Additionally, it was reported that CDs coordination with Fe^3+^ could increase the delocalization of π‐orbitals in the resulting CD assemblies due to π‐orbital bridging by the d‐orbitals of the Fe^3+^.^[^ ^35^ ^]^ We speculate that this mechanism also contributes to the broadening of HA‐CDs̕ absorption spectrum into the NIR region.

Notably, the FTIR and XPS characterizations confirmed the presence of numerous hydrophilic groups on the surface of HA‐CDs, and the small water contact angle (≈50°, Figure S15, Supporting Information) further validated the good hydrophilicity of HA‐CDs, which was beneficial for water wetting, allowing the water molecules to fully interact with the HA‐CDs. At the same time, the porous architectures of HA‐CDs with large amounts of mesopores (most above 20 nm in size) could increase the specific surface area (71.61 m^2^ g^−1^, Figure S16 and Table S4, Supporting Information) in contact with water and facilitate vapor diffusion. When compared to the commonly used carbon‐based photothermal materials, such as graphene and carbon nanotubes, the HA‐CDs are facilely synthesized without the need for high‐temperature calcination and simultaneously achieve efficient full‐solar spectrum absorption, water resistance, good hydrophilicity due to the surface‐retained hydrophilic functional groups, which provides a unique low‐cost and environmentally friendly advantage as a photothermal material for interfacial water evaporation.

### 2.2 Development of Planar Photothermal Converter based on HA‐CDs

**Figure 3:**
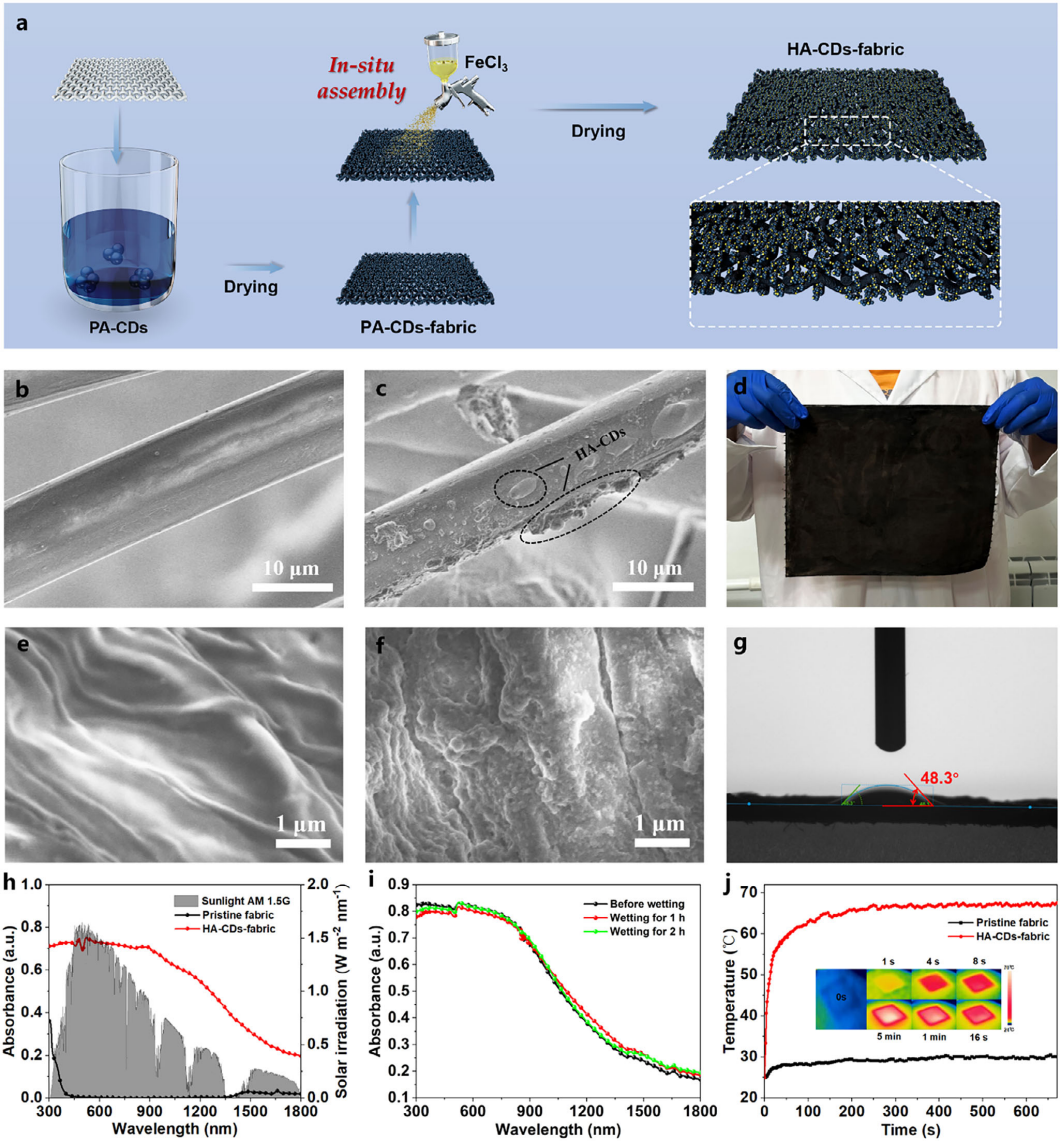
a) Illustration of fabrication procedure of HA‐CDs‐fabric. b,e) SEM images of pristine non‐woven fabric at different magnifications. c,f) SEM images of HA‐CDs‐fabric at different magnifications. d) Photograph of a large‐sized HA‐CDs‐fabric. g) The contact angle of water on the surface of HA‐CDs‐fabric. h) Absorption spectra of pristine non‐woven fabric, HA‐CDs‐fabric, and the solar radiation spectrum. i) Absorption spectra of HA‐CDs‐fabric before and after one hour and two hours of wetting. j) Photothermal conversion behaviors of pristine non‐woven fabric and HA‐CDs‐fabric under simulated 1 kW m^−2^ solar irradiation (Insets: IR thermal images of the HA‐CDs‐fabric at different times during the heating process).

Due to the facile and environmentally friendly assembling process, we developed in situ preparation of HA‐CDs on a non‐woven fabric (spunlaced non‐woven fabric composed of 55% cellulose and 45% polyester) matrix by dropwise addition of FeCl_3_ solution to the PA‐CDs loaded non‐woven fabric (**Figure** **3**a), with the color changed from deep blue for PA‐CDs loaded non‐woven fabric (PA‐CDs‐fabric) to black for HA‐CDs loaded non‐woven fabric (HA‐CDs‐fabric). The homogeneous color distribution indicated the uniform loading of HA‐CDs on the fabric. As shown in the SEM images (Figure 3b,c; Figure S17, Supporting Information), microclusters were deposited on the fibers of non‐woven fabric after the in situ formation of HA‐CDs. Surface observations also revealed that the pristine non‐woven fabric had a smooth surface with strip‐shaped wrinkles (Figure 3e), whereas the HA‐CDs‐fabric exhibited an uneven surface with numerous clusters attached (Figure 3f). The HA‐CDs‐fabric was facile to prepare and its feasibility for large‐area fabrication was demonstrated (Figure 3d). The HA‐CDs‐fabric displayed strong and broad absorption (300−1800 nm) that matched the solar spectrum compared to the pristine non‐woven fabric with absorption at wavelengths shorter than 450 nm (Figure 3h). The photothermal performance of HA‐CDs‐fabric was evaluated under simulated 1 kW m^−2^ solar irradiation. Within the very first minute, the surface temperature of HA‐CDs‐fabric rose rapidly from 25 to 60 °C and could be maintained at ≈68 °C for an extended period, and as a contrast, the temperature of pristine non‐woven fabric only reached a maximum of 30 °C under the same condition (Figure 3j). At the same time, the HA‐CDs‐fabric exhibited good wetting properties, with the contact angles dropping from 58.1° for the pristine fabric (Figure S18a, Supporting Information) to 48.3° for the HA‐CDs‐fabric (Figure 3g; Figure S18b, Supporting Information). To assess the water resistance, both PA‐CDs‐fabric and HA‐CDs‐fabric were soaked in water for 1 h, and it was observed that the PA‐CDs rapidly dissolved from the non‐woven fabric, while HA‐CDs remained essentially intact on the fabric (Figure S19, Supporting Information), demonstrating the good water resistance of the HA‐CDs‐fabric. Moreover, no apparent changes in the absorption spectra of the HA‐CDs‐fabric were detected during two hours of wetting (Figure 3i). The broad absorption, superior photothermal capacity, improved water resistance, and wetting capability enable HA‐CDs‐fabric to be a promising 2D evaporator for high‐performance water evaporation.

### 2.3 Solar Energy Applications of Planar Photothermal Converter based on HA‐CDs‐Fabric

**Figure 4:**
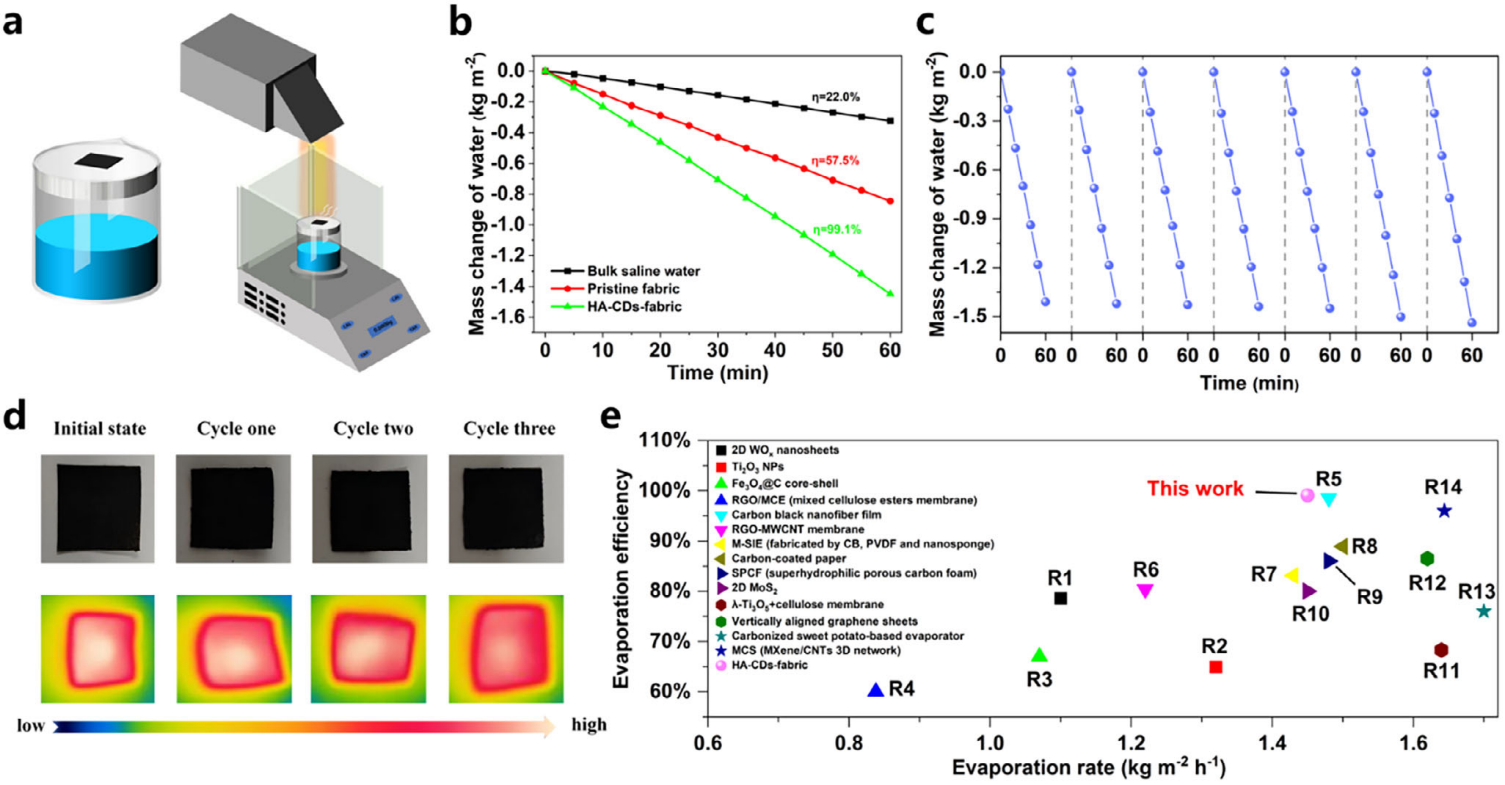
a) Schematic diagram of the interfacial water evaporation system equipped with HA‐CDs‐fabric as a solar absorber. b) Mass loss curves for bulk saline water, evaporation systems with pristine fabric evaporator, and HA‐CDs‐fabric evaporator for saline water during 1 h of simulated 1 kW m^−2^ solar irradiation. c) Mass loss curves of HA‐CDs‐fabric evaporator for seven cycles of water evaporation experiments (each cycle of evaporation experiment was conducted for 1 h under simulated 1 kW m^−2^ solar irradiation). d) Photographs and IR thermal images of HA‐CDs‐fabric at the end of each cycle of the evaporation experiment. e) Steam generation performances of HA‐CDs‐fabric and previously reported materials under 1 kW m^−2^ solar irradiation (the data of R1‐R10 and R12‐R14 from Refs. [41, 42, 43, 44, 45, 46, 47, 48, 49, 50, 51, 52, 53], R11 from Ref. [5]).

As depicted in **Figure** **4**a; Figure S20a‒c (Supporting Information), an interfacial water evaporation system was designed by employing HA‐CDs‐fabric as the 2D solar absorber and a non‐woven fabric strip as the water delivery channel, which was supported by the insulating expanded polyethylene (EPE) frame. Under 1 kW m^−2^ simulated solar irradiation, the mass reduction of saline water (3.5 wt.% NaCl solution) was monitored in real‐time and the evaporation of water with HA‐CDs‐fabric absorber was significantly accelerated with the evaporation rate measured to be 1.45 kg m^−2^ h^−1^ (detailed calculations refer to Supporting Information), which was prominently 4.5 and 1.7 times higher than those of bulk saline water (0.32 kg m^−2^ h^−1^) and saline water with pristine non‐woven fabric absorber (0.85 kg m^−2^ h^−1^), as shown in Figure 4b. We further estimated the durability of the HA‐CDs‐fabric‐based evaporator through seven rounds of water evaporation experiments (each lasting 1 h) and found no obvious change in the evaporation rate, which remained as high as 1.41−1.54 kg m^−2^ h^−1^ (Figure 4c). Notably, the seven cycles of water evaporation did not result in leakage of HA‐CDs, indicating the long‐term stability of the evaporator, in consistent with the above results (Figure 3i; Figure S19, Supporting Information). The minor rises in evaporation rate as the number of experimental cycles increased were attributed to the increased ambient temperature. In contrast, employing PA‐CDs‐fabric as the solar absorber (with UV–vis‐NIR absorption as exhibited in Figure S21a, Supporting Information) resulted in a significant leakage problem during the water evaporation process. Under the same conditions of three cycles of water evaporation, the water evaporation system with PA‐CDs‐fabric exhibited a constantly declining evaporation rate from 1.32 to 1.03 kg m^−2^ h^−1^ (Figure S21b,c, Supporting Information). This again demonstrated that the second‐step assembly could effectively improve the water‐resistance of the assemblies. To avoid the accumulation of mass salt residues on the surface of the evaporator, the length of the non‐woven fabric strips in two sides was adjusted and shown in Figure S20b (Supporting Information). The long fabric strip on the left side was immersed in 3.5 wt.% NaCl solution and the short fabric strip on the right side were placed above the liquid surface. As illustrated in Figure S22a (Supporting Information), a continuous water supply from the left side long fabric strip could wash away a large amount of salt residues from the evaporator, with the water and dissolved salts flowing back into the vessel along the right side short fabric strip. This method prevented the accumulation of excessive salts in the center of the evaporator, which could otherwise hinder the subsequent evaporation performance.^[^ ^40^ ^]^ After three cycles of evaporation, the evaporator surface remained largely free of salt residues, with only a minimal amount of salt residues detected at the right edge (Figure 4d; Figure S22b, Supporting Information). Meanwhile, the IR thermal images of the evaporator captured at the end of each heating cycle revealed a tiny decline in temperature at the right edge of the evaporator (Figure 4d) after the third evaporation round, which was caused by the presence of a minor amount of salt residues on the right side. Because of the good water resistance, salts on the HA‐CDs‐fabric could be rinsed away easily, allowing for continuous use of the evaporator. In sharp contrast, when salts were rinsed off the PA‐CDs‐fabric, both the salts and the PA‐CDs were washed away at the same time (Figure S23, Supporting Information). As depicted in Figure 4e, the 2D evaporator made from HA‐CDs‐fabric exhibited promising water evaporation performance relative to other reported evaporators utilizing different photothermal materials.^[^ ^5^, ^41^, ^42^, ^43^, ^44^, ^45^, ^46^, ^47^, ^48^, ^49^, ^50^, ^51^, ^52^, ^53^ ^]^

**Figure 5:**
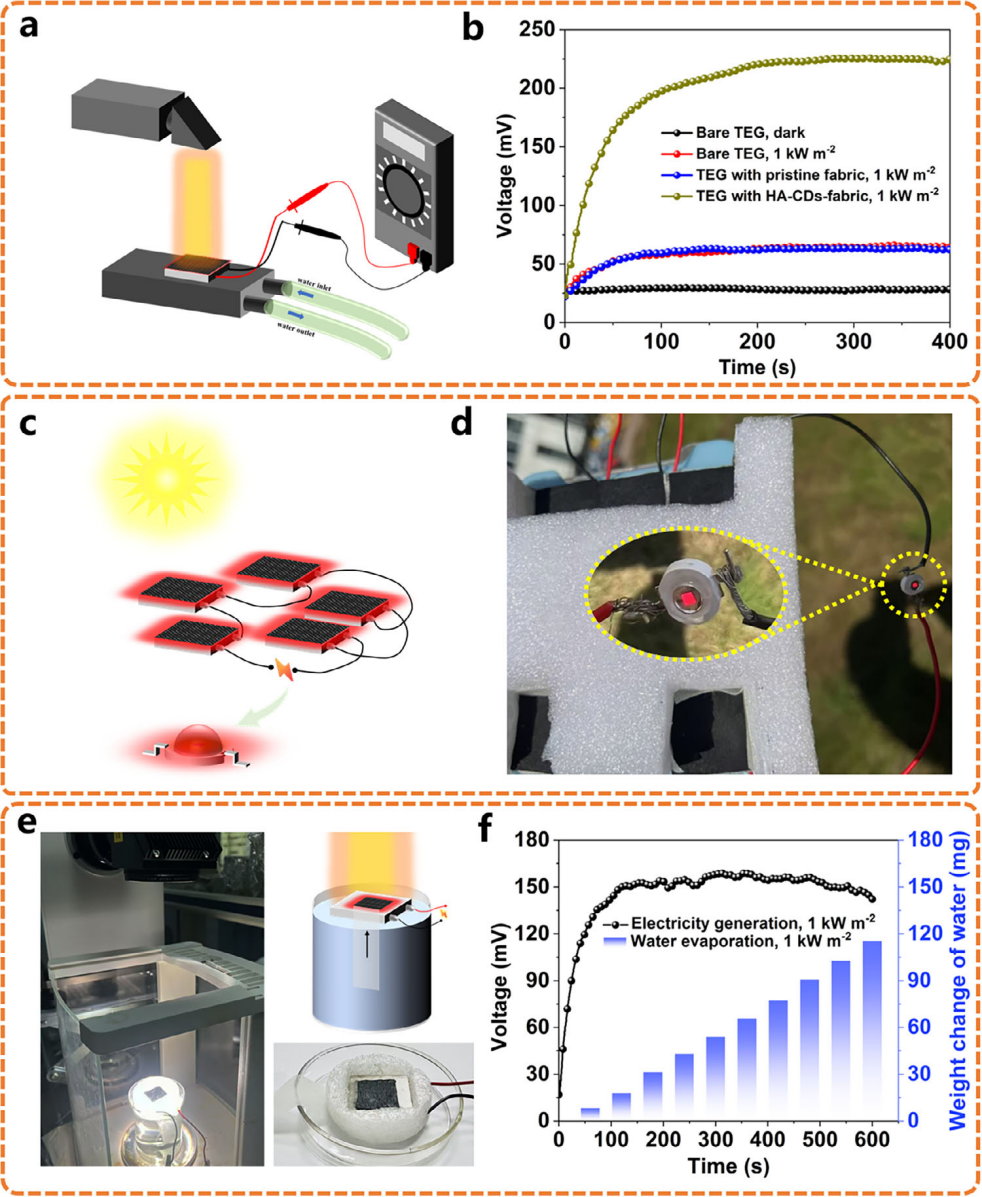
a) Schematic illustration of thermoelectric module for power generation. b) Open circuit voltage of TEGs with different materials as the hot side. c) Schematic design and application of an outdoor thermoelectric device. d) Photograph of an LED lamp illuminated by the thermoelectric device outdoors (Inset: glowing LED lamp with 1.5 V rated voltage). e) Schematic illustration and photograph of an integrated device for water evaporation and electricity generation. f) Open‐circuit voltages and mass of evaporated water of the water‐electricity cogeneration system under 1 kW m^−2^ solar irradiation.

In order to make full use of solar energy, the waste heat from photothermal conversion can be utilized by thermoelectric devices to generate electricity (**Figure** **5**a). According to the Seebeck effect, the temperature difference between two dissimilar electrical conductors or semiconductors can generate an electric potential,^[^ ^17^ ^]^ converting thermal energy directly into electrical energy with the benefits of long service life, low maintenance, portability, noiseless, and vibrationless. In this regard, the remarkable photothermal conversion capacity of HA‐CDs allows them to be employed for not only water evaporation but also solar‐driven electricity generation. A thermoelectric device was fabricated using a thermoelectric generator (TEG, TEC1‐12701K34) with HA‐CDs‐fabric solar absorber on the hot side and the cool side placed on a cooling unit with circulating condensate water (Figure S24a,b, Supporting Information). Under simulated 1 kW m^−2^ solar irradiation, the output voltage of the TEG with HA‐CDs‐fabric as hot side increased rapidly, reaching a maximum 225 mV in 4 min and maintaining the maximum voltage for the following time, which showed a significant enhancement of ≈160 mV compared to both the bare TEG and TEG with the hot side covered by pristine non‐woven fabric (Figure 5b; Figure S24c, Supporting Information). Furthermore, we performed thermoelectric experiment outdoors under sunlight irradiation by connecting five of the HA‐CDs‐fabric‐based TEGs in series and with cooling gels sticking to cool sides. The experiment was conducted as depicted in Figure 5c, when the TEGs were exposed to sunlight, the output voltage could light up an LED lamp with 1.5 V rated voltage (Figure 5d). Based on the aforementioned findings, an integrated system combining solar‐driven water evaporation and thermoelectric generation was further developed. As illustrated in Figure 5e, the HA‐CDs‐fabric (2 cm×2 cm) was coated on the hot side of the TEG, with a fabric strip attached to the edge as a water channel. The TEG floated on the surface of the water as a cool side, supported by a EPE frame. The co‐generation system could simultaneously evaporate 115 mg water and produce a maximum voltage of ≈155 mV for power generation under 10 min of simulated 1 kW m^−2^ solar irradiation (Figure 5f). The successful solar‐driven water evaporation, thermoelectric power generation, and water‐electricity cogeneration highlighted the promising application potential of HA‐CDs in efficient solar energy capture and conversion.

## 3 Conclusion

To conclude, hierarchical assemblies of CDs (HA‐CDs) have been smartly constructed by two steps of assembly, including the formation of primary assemblies of CDs (PA‐CDs) with broadband absorption for the first step and complexation of PA‐CDs with Fe^3+^ for the second step. The HA‐CDs exhibit full‐solar‐spectrum absorption (300−1800 nm), superior photothermal conversion capacity with efficiency of 84% under solar irradiation, improved stability, good water resistance, surface hydrophilicity, and ease of in situ preparation on fabric substrates. Benefiting from these advantages, HA‐CDs in situ coated fabric has been successfully used in solar‐driven interfacial water evaporation, thermoelectric generator, and water‐electricity cogeneration, demonstrating remarkable performance. This work highlights the appealing structural and optical properties and promising applications of CD hierarchical assemblies in solar energy capture and conversion. It provides a novel, practical, and environmentally friendly design approach for developing high‐performance carbon‐based photothermal materials and related devices for solar energy applications.

## 4 Experimental Section

### Synthesis of F‐CDs and PA‐CDs

F‐CDs were synthesized based on a hydrothermal method reported in a previous work,^[^ ^28^ ^]^ all processes are as follows: 3 g citric acid and 6 g urea were dissolved in 24 mL ultrapure water, and then the mixtures were transferred to a 50 mL Teflon‐lined stainless‐steel autoclave and heated at 160 °C for 4 h. The resultant solution was centrifugated at 8000 rpm for 5 min to remove the precipitate. The solution was freeze‐dried to obtain the yellow powders of F‐CDs. Subsequently, the F‐CDs were placed in a humid environment for two weeks until they completely transformed into black. Finally, the black powders were diffused and dialyzed in ultrapure water using a dialysis bag with a molecule weight cutoff of 1000 Da, and freeze‐dried to obtain the purified deep blue powders of PA‐CDs.

### Synthesis of HA‐CDs

PA‐CDs were dissolved in ultrapure water to obtain a solution with a concentration of 25 mg mL^−1^. Ferric chloride powders were dissolved in ultrapure water to obtain a solution with a concentration of 0.8 mol L^−1^. 200 µL ferric chloride solution was mixed with 1 mL PA‐CDs solution under magnetic stirring and then the mixed solution was centrifugated at 5000 rpm for 5 min to obtain the precipitates. The precipitates were continuously washed by ultrapure water for further purification. Finally, the precipitates were freeze‐dried to obtain the black powders of HA‐CDs.

### Fabrication of PA‐CDs‐Fabric and HA‐CDs‐Fabric

Non‐woven fabric with a square shape was immersed in PA‐CDs solution and then dried in the oven. This process was repeated continuously until ≈25 mg PA‐CDs were loaded on the fabric to obtain the PA‐CDs‐fabric. Subsequently, 200 µL ferric chloride solution (0.8 mol L^−1^) was dropped onto PA‐CDs‐fabric and dried, and then the fabric was immersed in ultrapure water to remove the unreacted materials and dried to obtain the HA‐CDs‐fabric.

## Conflict of Interest

The authors declare no conflict of interest.

## Author Contributions

L.L., Y.Q., M.L., H.B., R.Z., and S.Q. performed the experiments, interpreted the data, and generated the figures. D.L., T.J., S.Q., and W.Z. supervised the experiments, wrote the manuscript, and conceived the project.

## Supporting information



Supporting Information

## Data Availability

The data that support the findings of this study are available from the corresponding author upon reasonable request.

## References

[1] P. Tao , G. Ni , C. Song , W. Shang , J. Wu , J. Zhu , G. Chen , T. Deng , Nat. Energy 2018, 3, 1031.

[2] D. Kraemer , B. Poudel , H.‐P. Feng , J. C. Caylor , B. Yu , X. Yan , Y. Ma , X. Wang , D. Wang , A. Muto , K. McEnaney , M. Chiesa , Z. Ren , G. Chen , Nat. Mater. 2011, 10, 532. 21532584 10.1038/nmat3013

[3] F. Zhao , Y. Guo , X. Zhou , W. Shi , G. Yu , Nat. Rev. Mater. 2020, 5, 388.

[4] X. Cui , Q. Ruan , X. Zhuo , X. Xia , J. Hu , R. Fu , Y. Li , J. Wang , H. Xu , Chem. Rev. 2023, 123, 6891. 37133878 10.1021/acs.chemrev.3c00159 PMC10273250

[5] B. Yang , Z. Zhang , P. Liu , X. Fu , J. Wang , Y. Cao , R. Tang , X. Du , W. Chen , S. Li , H. Yan , Z. Li , X. Zhao , G. Qin , X.‐Q. Chen , L. Zuo , Nature 2023, 622, 499. 37704732 10.1038/s41586-023-06509-3

[6] Y. Li , Y. Shi , H. Wang , T. Liu , X. Zheng , S. Gao , J. Lu , Carbon Energy 2023, 5, e331.

[7] L. Wang , Y. Wang , T. Xu , H. Liao , C. Yao , Y. Liu , Z. Li , Z. Chen , D. Pan , L. Sun , M. Wu , Nat. Commun. 2014, 5, 5357. 25348348 10.1038/ncomms6357

[8] C. Xia , S. Zhu , T. Feng , M. Yang , B. Yang , Adv. Sci. 2019, 6, 1901316. 10.1002/advs.201901316 PMC6891914 31832313

[9] X. Li , L. Yu , M. He , C. Chen , Z. Yu , S. Jiang , Y. Wang , L. Li , B. Li , G. Wang , A. Shen , J. Fan , BMEMat 2023, 1, e12045.

[10] L. Dordevic , F. Arcudi , M. Cacioppo , M. Prato , Nat. Nanotechnol. 2022, 17, 112. 35173327 10.1038/s41565-021-01051-7

[11] B. Wang , S. Lu , Matter 2022, 5, 110.

[12] Y. Shi , W. Su , F. Yuan , T. Yuan , X. Song , Y. Han , S. Wei , Y. Zhang , Y. Li , X. Li , L. Fan , Adv. Mater. 2023, 35, 2210699. 10.1002/adma.202210699 36959751

[13] Z. Kang , B. Yang , M. Prato , Small 2023, 19, 2304703. 10.1002/smll.202304703 37533137

[14] Y. Ding , S. Liu , L. Yang , G. Du , J. Wan , Z. Chen , S. Li , Small 2024, 20, 2401942. 10.1002/smll.202401942 38593325

[15] Q. Hou , C. R. Xue , N. Li , H. Q. Wang , Q. Chang , H. T. Liu , J. L. Yang , S. L. Hu , Carbon 2019, 149, 556.

[16] C. Liu , J. Su , Q. Chang , Y. Li , J. Yang , S. Hu , Sol. RRL 2022, 6, 2200170.

[17] J. Wan , J. Wang , H. Guo , K. Wan , X. Zhao , J. Li , S. Li , Z. Chen , S. Liu , K. Zhang , Matter 2022, 5, 2864.

[18] M. A. Sk , A. Ananthanarayanan , L. Huang , K. H. Lim , P. Chen , J. Mater. Chem. C 2014, 2, 6954.

[19] Z. Tian , X. Zhang , D. Li , D. Zhou , P. Jing , D. Shen , S. Qu , R. Zboril , A. L. Rogach , Adv. Opt. Mater. 2017, 5, 1700416.

[20] X. Bao , Y. Yuan , J. Chen , B. Zhang , D. Li , D. Zhou , P. Jing , G. Xu , Y. Wang , K. Holá , D. Shen , C. Wu , L. Song , C. Liu , R. Zbořil , S. Qu , Light:Sci. Appl. 2018, 7, 91. 30479757 10.1038/s41377-018-0090-1 PMC6249234

[21] B. Geng , W. Shen , F. Fang , H. Qin , P. Li , X. Wang , X. Li , D. Pan , L. Shen , Carbon 2020, 162, 220.

[22] Q. Wang , T. Zhang , Q. Cheng , B. Wang , Y. Liu , G. Xing , Z. Tang , S. Qu , Adv. Funct. Mater. 2024, 34, 2402976.

[23] D. Li , P. Jing , L. Sun , Y. An , X. Shan , X. Lu , D. Zhou , D. Han , D. Shen , Y. Zhai , S. Qu , R. Zbořil , A. L. Rogach , Adv. Mater. 2018, 30, 1705913. 10.1002/adma.201705913 29411443

[24] D. Li , C. Liang , E. V. Ushakova , M. Sun , X. Huang , X. Zhang , P. Jing , S. J. Yoo , J.‐G. Kim , E. Liu , W. Zhang , L. Jing , G. Xing , W. Zheng , Z. Tang , S. Qu , A. L. Rogach , Small 2019, 15, 1905050. 10.1002/smll.201905050 31721434

[25] D. Li , Y. Qu , X. Zhang , W. Zheng , A. L. Rogach , S. Qu , Chem. Eng. J. 2023, 454, 140069.

[26] B. Bartolomei , M. Sbacchi , C. Rosso , A. Günay‐Gürer , L. Zdražil , A. Cadranel , S. Kralj , D. M. Guldi , M. Prato , Angew. Chem., Int. Ed. 2024, 63, e202316915. 10.1002/anie.202316915 38059678

[27] T. Liang , E. Liu , M. Li , E. V. Ushakova , S. V. Kershaw , A. L. Rogach , Z. Tang , S. Qu , ACS Nano 2021, 15, 1579. 33356126 10.1021/acsnano.0c09053

[28] D. Li , D. Han , S.‐N. Qu , L. Liu , P.‐T. Jing , D. Zhou , W.‐Y. Ji , X.‐Y. Wang , T.‐F. Zhang , D.‐Z. Shen , Light:Sci. Appl. 2016, 5, e16120. 30167175 10.1038/lsa.2016.120 PMC6059947

[29] J. Wang , C. Xu , A. M. Nilsson , D. L. A. Fernandes , M. Strömberg , J. Wang , G. A. Niklasson , Adv. Opt. Mater. 2019, 7, 1801315.

[30] S. Qu , X. Wang , Q. Lu , X. Liu , L. Wang , Angew. Chem., Int. Ed. 2012, 51, 12215. 10.1002/anie.201206791 23109224

[31] Y. Hu , Z. Chen , F. Lai , J. Li , J. Mater. Sci. 2019, 54, 8627.

[32] S. Jain , J. Shah , N. S. Negi , C. Sharma , R. K. Kotnala , Int. J. Energy Res. 2019, 43, 4743.

[33] A. P. Grosvenor , B. A. Kobe , M. C. Biesinger , N. S. McIntyre , Surf. Interface Anal. 2004, 36, 1564.

[34] S. Zhu , Q. Meng , L. Wang , J. Zhang , Y. Song , H. Jin , K. Zhang , H. Sun , H. Wang , B. Yang , Angew. Chem., Int. Ed. 2013, 52, 3953. 10.1002/anie.201300519 23450679

[35] Z.‐B. Qu , W.‐J. Feng , Y. Wang , F. Romanenko , N. A. Kotov , Angew. Chem., Int. Ed. 2020, 59, 8542. 10.1002/anie.201908216 31475420

[36] C.‐L. Shen , Q. Lou , K.‐K. Liu , G.‐S. Zheng , R.‐W. Song , J.‐H. Zang , L. Dong , C.‐X. Shan , Carbon 2023, 213, 118217.

[37] J. Liu , Y. Cui , Y. Pan , Z. Chen , T. Jia , C. Li , Y. Wang , Angew. Chem., Int. Ed. 2022, 61, e202117087. 10.1002/anie.202117087 35075755

[38] B. Lü , Y. Chen , P. Li , B. Wang , K. Müllen , M. Yin , Nat. Commun. 2019, 10, 767. 30770818 10.1038/s41467-019-08434-4 PMC6377642

[39] Y. Wang , Y. Ji , Y. Yang , Z. Chen , H. Sun , X. Wang , Z. Zou , H. Huang , ACS Energy Lett. 2024, 9, 336.

[40] M. Ye , N. Tao , X. Zhou , X. Wang , W. Jin , T. Zhang , X. Liu , Sep. Purif. Technol. 2023, 311, 123201.

[41] I. Ibrahim , D. H. Seo , A. M. McDonagh , H. K. Shon , L. Tijing , Desalination 2021, 500, 114853.

[42] J. Wang , Y. Li , L. Deng , N. Wei , Y. Weng , S. Dong , D. Qi , J. Qiu , X. Chen , T. Wu , Adv. Mater. 2017, 29, 1603730. 10.1002/adma.201603730 27862379

[43] R. Chen , K. Zhu , Q. Gan , Y. Yu , T. Zhang , X. Liu , M. Ye , Y. Yin , Mater. Chem. Front. 2017, 1, 2620.

[44] G. Wang , Y. Fu , X. Ma , W. Pi , D. Liu , X. Wang , Carbon 2017, 114, 117.

[45] R. Zhang , Y. Zhou , B. Xiang , X. Zeng , Y. Luo , X. Meng , S. Tang , Adv. Mater. Interfaces 2021, 8, 2101160.

[46] Y. Wang , C. Wang , X. Song , S. K. Megarajan , H. Jiang , J. Mater. Chem. A 2018, 6, 963.

[47] M. Xia , J. Wei , Z. Han , Q. Tian , C. Xiao , Q.‐M. Hasi , Y. Zhang , L. Chen , Mater. Today Energy 2022, 25, 100959.

[48] H. Song , Y. Liu , Z. Liu , M. H. Singer , C. Li , A. R. Cheney , D. Ji , L. Zhou , N. Zhang , X. Zeng , Z. Bei , Z. Yu , S. Jiang , Q. Gan , Adv. Sci. 2018, 5, 1800222. 10.1002/advs.201800222 PMC6096986 30128237

[49] C. Wang , J. Wang , Z. Li , K. Xu , T. Lei , W. Wang , J. Mater. Chem. A 2020, 8, 9528.

[50] X. Li , W. Xu , M. Tang , L. Zhou , B. Zhu , S. Zhu , J. Zhu , Chemistry 2016, 113, 13953. 10.1073/pnas.1613031113 PMC5150409 27872280

[51] P. Zhang , J. Li , L. Lv , Y. Zhao , L. Qu , ACS Nano 2017, 11, 5087. 28423271 10.1021/acsnano.7b01965

[52] W. Wang , Z. Tian , X. Huan , Y. Li , P. Hu , Energy Technol. 2024, 12, 2300671.

[53] Y. Jin , X. Gong , Y. He , H. Wang , S. Li , J. Liu , Sol. RRL 2023, 7, 2300548.

