# Supplementary material for: Hierarchical Assembly of Carbon Dots with Full‐Solar‐Spectrum Absorption for Solar Energy Applications
Source: Adv Sci (Weinh). 2025 Mar 17;12(18):2417457. doi: 10.1002/advs.202417457 (PMC12079387; doi:10.1002/advs.202417457)
Supplement: Supplementary file 1 — Supporting Information [file ADVS-12-2417457-s001.docx]

Supporting Information

**Hierarchical Assembly of Carbon Dots with Full-Solar-Spectrum Absorption for Solar Energy Applications**

Lei Li, Di Li^*^, Yanfei Qu, Ruoyu Zhang, Shuo Qi, Mengyao Liu, Haohao Bi, Tao Jia^*^, Songnan Qu^*^, and Weitao Zheng^*^

Lei Li, Di Li, Yanfei Qu, Mengyao Liu, Haohao Bi & Weitao Zheng

Key Laboratory of Automobile Materials, College of Materials Science and Engineering, Jilin University, Changchun, 130012 P. R. China

Ruoyu Zhang, Shuo Qi & Tao Jia

Key Laboratory of Forest Plant Ecology, Ministry of Education, Engineering Research Center of Forest Bio-Preparation, College of Chemistry, Chemical Engineering and Resource Utilization, Northeast Forestry University, Harbin 150040, China

Songnan Qu

Joint Key Laboratory of the Ministry of Education, Institute of Applied Physics and Materials Engineering, University of Macau, Avenida da Universidade, Taipa, Macau SAR, 999078, P. R. China

**Table of contents**

1. Materials

2. Basic characterization techniques

3. Production repeatability of HA-CDs

4. Measurement and calculation of the photothermal conversion efficiency under simulated 1 kW m^-2^ solar irradiation

5. Measurement of solar-driven interfacial water evaporation and calculation of the evaporation rate and efficiency for solar to vapor generation

6. Optical properties of F-CDs and PA-CDs

7. TEM images of F-CDs, PA-CDs and HA-CDs

8. Raman spectrum of HA-CDs

9. XPS spectra of PA-CDs and HA-CDs

10. FT-IR spectra of PA-CDs and HA-CDs

11. Zeta potentials of PA-CDs and HA-CDs

12. Absorption of PA-CDs and HA-CDs in solids

13. Water resistance of PA-CDs and HA-CDs

14. Stability of HA-CDs

15. Photothermal properties of HA-CDs under 1 kW m^-2^ solar irradiation

16. Photothermal conversion properties of HA-CDs under irradiation of 1064 nm laser

17. Water wetting capability of HA-CDs

18. BET analysis of HA-CDs

19. Microstructure of pristine non-woven fabric

20. Water wetting capability of pristine non-woven fabric and HA-CDs-fabric

21. Water resistance evaluation for PA-CDs-fabric and HA-CDs-fabric

22. Experimental platform of interfacial water evaporation system employing pristine non-woven fabric as the evaporator

23. Water evaporation performance of PA-CDs-fabric evaporator

24. Illustration of salt precipitation of the evaporation system employing HA-CDs-fabric evaporator

25. Salts removal from the surface of the evaporator by water rinsing

26. Experimental platform for thermoelectric generation

**1. Materials**

Citric acid (ACS, ≥99.5%), urea (AR, 99%), and iron (III) chloride anhydrous (AR, 99%) were purchased from Aladdin. Water was obtained from the laboratory using Keruifeng Experimental UltraPure Water System. All chemicals were used as received without further purification.

**2. Basic characterization techniques**

SEM was measured on a JEOL JSM-6700F field emission scanning electron microscopy. TEM was performed on a FEI Tecnai-G2-F20 transmission electron microscopy at 200 kV. Raman spectrum was measured by LabRAM HR Evolution Raman spectrometer under excitation of 532 nm. FT-IR spectra were obtained on a Nicolet 6700 FT-IR spectrometer. XPS was performed on an ESCALAB MK I X-ray photoelectron spectrometer using Mg as the excitation source. Absorption spectra were obtained on Shimadzu UV3101PC and LAMBDA 1050+ UV-Vis-NIR Spectrometer. Contact angles were measured on dataphysics OCA50. Physical adsorption test was performed on ASAP2460 physisorption analyzer.

**3. Production repeatability of HA-CDs**

To verify the production repeatability, we first conducted three parallel synthesis experiments. The HA-CDs obtained from three independent syntheses exhibited essentially the same absorption spectra and morphologies (**Figure S1** and **S2**). To further investigate the scalability of the process, we conducted experiments to scale up and down the synthesis. When the mass of the starting materials was increased and decreased by twice at a constant concentration, the product mass increased and decreased by approximately twice accordingly (**Table S1**). These results demonstrate the good production repeatability.


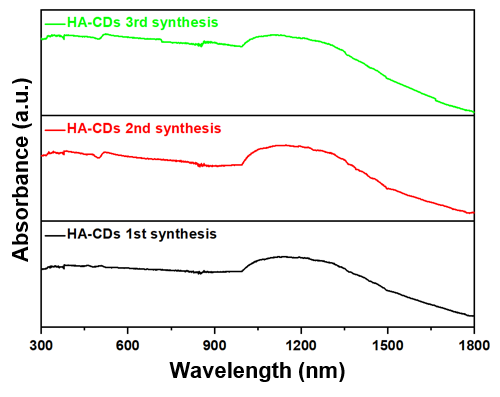


**Figure S1.** The absorption spectra of HA-CDs obtained from three independent synthesis experiments.


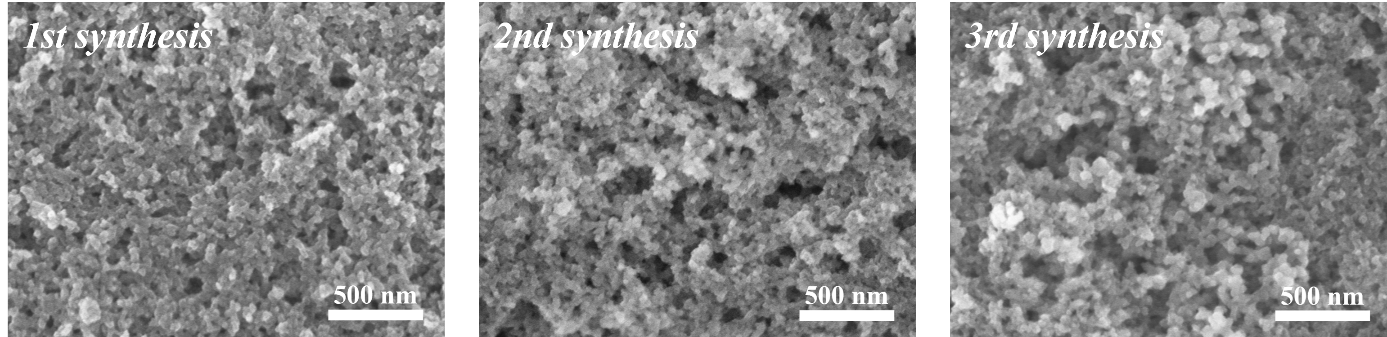


**Figure S2.** The SEM images of the HA-CDs obtained from three independent syntheses.

| **m_PA-CDs_ (mg)** | **m_HA-CDs_ (mg)** | **m_HA-CDs_/m_PA-CDs_ (%)** |
| --- | --- | --- |
| 12.5 | 7.2 | 57.6 |
| 25 | 14.7 | 58.8 |
| 25 | 15.3 | 61.2 |
| 25 | 15.5 | 62 |
| 50 | 32.8 | 65.6 |

**Table S1.** Comparison of the masses of HA-CDs obtained from three independent syntheses, as well as the masses of HA-CDs from the reactions with proportionally scaled-down and scaled-up reactants.

**4. Measurement and calculation of the photothermal conversion efficiency under simulated 1 kW m^-2^ solar irradiation^1^**

The external photothermal efficiency of HA-CDs was determined using the following equation.

$\eta_{ext}=\frac{Q}{q}$ (1)

where Q is the thermal energy raised from irradiation and q is the power of direct solar irradiation on the material.

When HA-CDs were heated under ambient conditions by a specific solar irradiation, they absorb and convert solar light to heat, which was then dissipated by transmitting the energy to the surrounding air atmosphere. The continuum energy balance for this system can be written as follows:

$Q-Q_{surr}=c_{i}\times m_{i}\times\frac{dT(t)}{dt}$ (2)

in which $Q$ is the heat generated, $Q_{surr}$ is the heat dissipated, $c_{i}$ and $m_{i}$ are heat capacity and the sample mass, respectively, $T$ is temperature of the material, and $t$ is time. In this equation, $Q_{surr}$ can be considered as linearly proportional to temperature change as

$Q_{surr}=h\times s\times(T-T_{surr})$ (3)

in which $h$ refers to a heat transfer coefficient, $s$ is the surface area of the material for heat dissipation, and $T_{surr}$ is the surrounding temperature. As solar light irradiation can cause an increase in the temperature of materials, it also increases heat dissipation. Herein, for a particular solar irradiation power, the temperature of the materials will rise and eventually reach a maximum temperature *T_max_* at where the energy input and output are in equilibrium as

$Q=Q_{surr}^{max}=h\times s\times(T_{max}-T_{surr}^{max})$ (4)

Where the solar irradiation increases the surrounding temperature to its maximum equilibrium temperature $T_{surr}^{max}$.

In order to determine the energy conversion efficiency $\eta_{ext}$, the only unknown parameter would be *hs*, as the solar power *q* and the maximum temperature of materials can be obtained experimentally.

According to Eqs (2) and (3), the time-dependent temperature change function $T(t)$ can be described as

$\frac{dT(t)}{dt}=\frac{Q-Q_{surr}}{c_{i}\times m_{i}}=\frac{Q}{c_{i}\times m_{i}}-\frac{h\times s}{c_{i}\times m_{i}}\times(T\left( t \right)-T_{surr}(t))$ (5)

While $Q/{{(c}_{i}\times m_{i}})$ can be considered as the amount of heat input, ${hs}/{c_{i}\times m_{i}}$ can be recognized as the rate constant of the energy loss *w*,

$w=\frac{h\times s}{c_{i}\times m_{i}}$ (6)

The solar irradiation stopped after continuous heating. If the system has reached the equilibrium state, at time $t=0$, we would have $Q=0$ and $T(0)=T_{max}$, hence Eq. (5) will change to

$\frac{dT(t)}{dt}=-w\times(T\left( t \right)-T_{surr}(t))$ (7)

$T_{surr}\left( t \right)=\left( T_{surr}^{max}-T_{surr}^{0} \right)\times\exp\left( -w_{surr}\times t \right)+T_{surr}^{0}$ (8)

Where $w_{surr}$ is the rate constant of the energy transfer in the surrounding environment, similar to $w$, and $T_{surr}^{0}$ represents the stable room temperature. According to Eqs (7) and (8), we can get the expression of *T(t)*:

$T\left( t \right)=A\times\exp\left( -w\times t \right)+B\times exp\left( -w_{surr}\times t \right)+C$ (9)

in which

$A=T_{max}-B-C$, $B=\frac{w}{w-w_{surr}}\times\left( T_{surr}^{max}-T_{surr}^{0} \right)$, and $C=T_{surr}^{0}$

During the experiment, powders of HA-CDs were placed on a non-woven fabric in a EPE holder with a square groove with size of 0.6 cm×0.6 cm. The simulated 1.0 kW m^-2^ sunlight was used to irradiate the samples to make them reach the maximum surface temperature. The temperature of the samples was recorded by the infrared camera. The rate constant $w$ , $w_{surr}$ and $T_{surr}^{max}$ could be easily obtained by fitting the cooling line. The sample mass $m_{i}$ was weighed and $c_{i}$ was determined through the Differential Scanning Calorimetry (DSC) test. All the parameters and their corresponding values were listed in (**Table S2 and Figure S3**). The photothermal conversion efficiency $\eta_{ext}$ was calculated to be 84%.

| parameter | value |
| --- | --- |
| $T_{max}/℃$ | 55.5 |
| $T_{surr}^{max}/℃$ | 34.4 |
| $T_{surr}^{0}/℃$ | 26.8 |
| $w/s^{-1}$ | 1/5.84 |
| $w_{surr}/s^{-1}$ | 1/48.20 |
| $m_{i}/g$ | 0.005 |
| $c_{i}/J.g^{-1}℃^{-1}$ | 1.67 |

**Table S2.** Experimental parameters for photothermal conversion efficiency measurement.


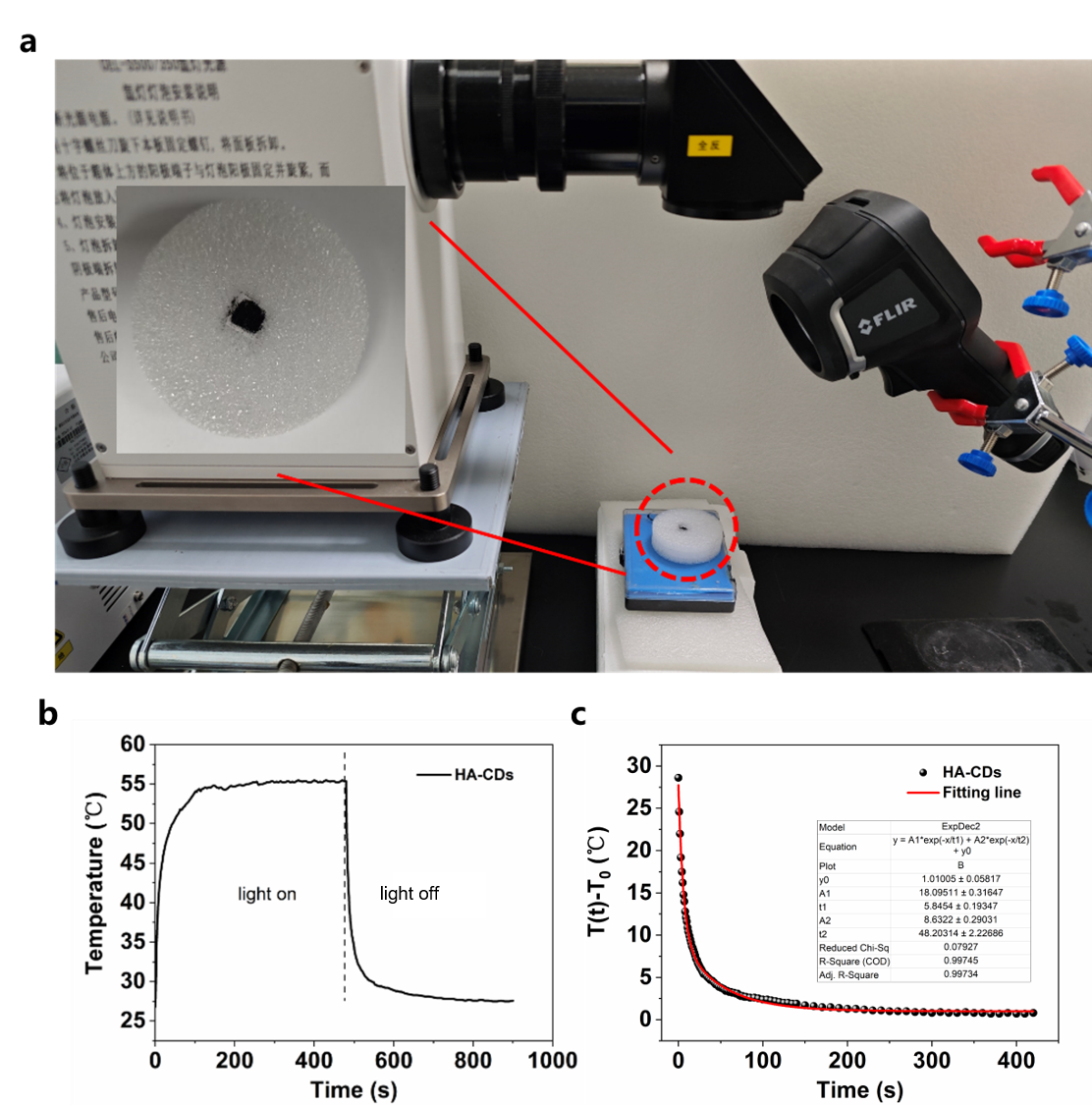


**Figure S3.** (a) Photograph of experiment setup. Inset: digital and IR photos of HA-CDs under 1 kW m^-2^ solar irradiation. (b) Temperature of HA-CD powders as a function of time with simulated 1 kW m^-2^ sunlight on and off. (c) The plot of temperature as a function of time after the light off.

**5. Measurement of solar-driven interfacial water evaporation and calculation of the evaporation rate and efficiency for solar to vapor generation^2^**

Solar-driven interfacial water evaporation was conducted under laboratory condition (room temperature: 25°C, humidity: 40%). Xenon lamp equipped with an AM 1.5G spectral filter was used as the solar simulator. An analytical balance was used for recording the mass change of water during the evaporation process. Solar power density was determined by the solar power meter. 3.5 wt.% sodium chloride solution was used for interfacial water evaporation test.

The conversion efficiency η of solar energy in photothermal water evaporation was calculated as the following equation.

$$\eta=\frac{\dot{m}h_{LV}}{C_{opt}P_{0}}$$

Where $\dot{m}$ refers to the mass flux of water, $\dot{m}=\frac{\Delta m}{S}$, $\Delta m$ is the mass of the evaporated water, $S$ is the area of the absorber, $h_{LV}$ refers to the total liquid vapor phase-change enthalpy $h_{LV}=Q+{\Delta h}_{vap}$, $Q$ is the energy provided to heat the system from the initial temperature to a final temperature, ${\Delta h}_{vap}$ is the latent heat of vaporization of water, $P_{0}$ is the nominal solar irradiation value of 1.0 kW m^-2^, and $C_{opt}$ represents the optical concentration ($C_{opt}=1$). The schematic for the vaporization enthalpy of the vapor is as follows.

$$Q=C_{liquid}\times\left( T-T_{0} \right)$$

$$\Delta h_{vap}=Q_{1}+\Delta h_{100}+Q_{2}$$

$$Q_{1}=C_{liquid}\times\left( 100-T \right)$$

$$Q_{2}=C_{vapor}\times\left( T-100 \right)$$

In this paper, , the specific heat capacity of liquid water ($C_{liquid}$) is a constant of 4.18 J g^-1^ ℃^-1^, the specific heat capacity of water vaper ($C_{vapor}$) is a constant of 1.865 J g^-1^ ℃^-1^, $\Delta h_{100}$ is the latent heat of vaporization of water at 100^o^C, taken to be 2260 kJ kg^-1^. $T_{0}$ represents the room temperature and $T$ represents the surface temperature of the absorber.

During 1 h irradiation, $\Delta m$ reached 0.145 g. The evaporation area is 1 ${cm}^{2}$($1 cm\times1 cm$). $\dot{m}=\frac{\Delta m}{S}=1.45 kg m^{-2}$. In this system, the temperature of the illuminated absorber is higher than the environment, thus it is unnecessary to deduct the dark evaporation.^3^

As a result, the evaporation rate and efficiency of HA-CDs-fabric were 1.45 $kg m^{-2}h^{-1}$ and 99.1% as shown in **Table S3**.

| **Time/**min | **T/**^o^C | **T_0_/**^o^C | **ṁ**/kg m^-2^ | **Q/**kJ kg^-1^ | **∆h_vap_/**kJ kg^-1^ | **Q_1_/**kJ kg^-1^ | **Q_2_/**kJ kg^-1^ | **H_LV_/** kJ kg^-1^ | **Efficiency/**% |
| --- | --- | --- | --- | --- | --- | --- | --- | --- | --- |
| 60 | 36.5 | 23.4 | 1.449 | 54.758 | 2407.003 | 265.43 | -118.428 | 2461.761 | 99.1 |

**Table S3.** Experimental parameters for the calculation of evaporation efficiency.

**6. Optical properties of F-CDs and PA-CDs**

**
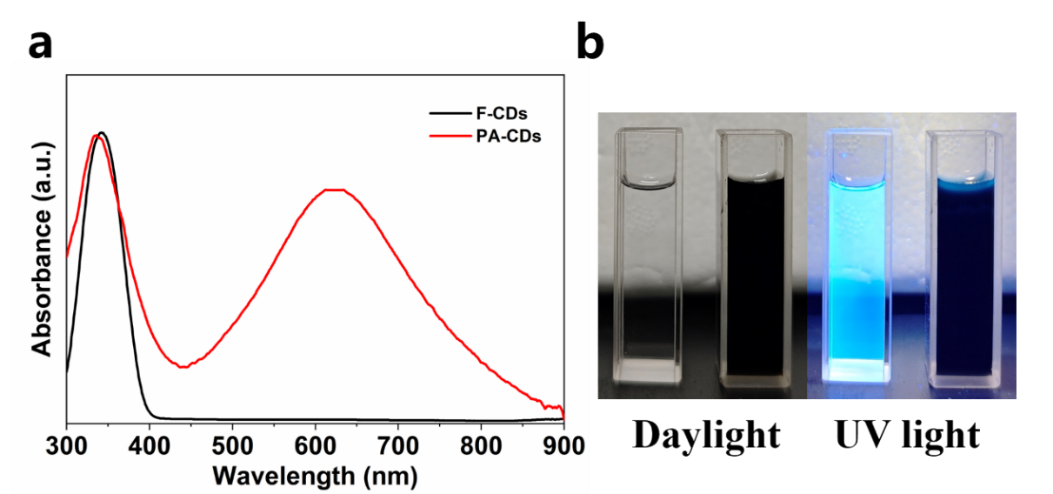
**

**Figure S4.** (a) Absorption spectra of F-CDs and PA-CDs diffused in water. (b) Digital photos of F-CDs and PA-CDs solution under daylight and UV light irradiation.

**7. TEM images of F-CDs, PA-CDs and HA-CDs**

**
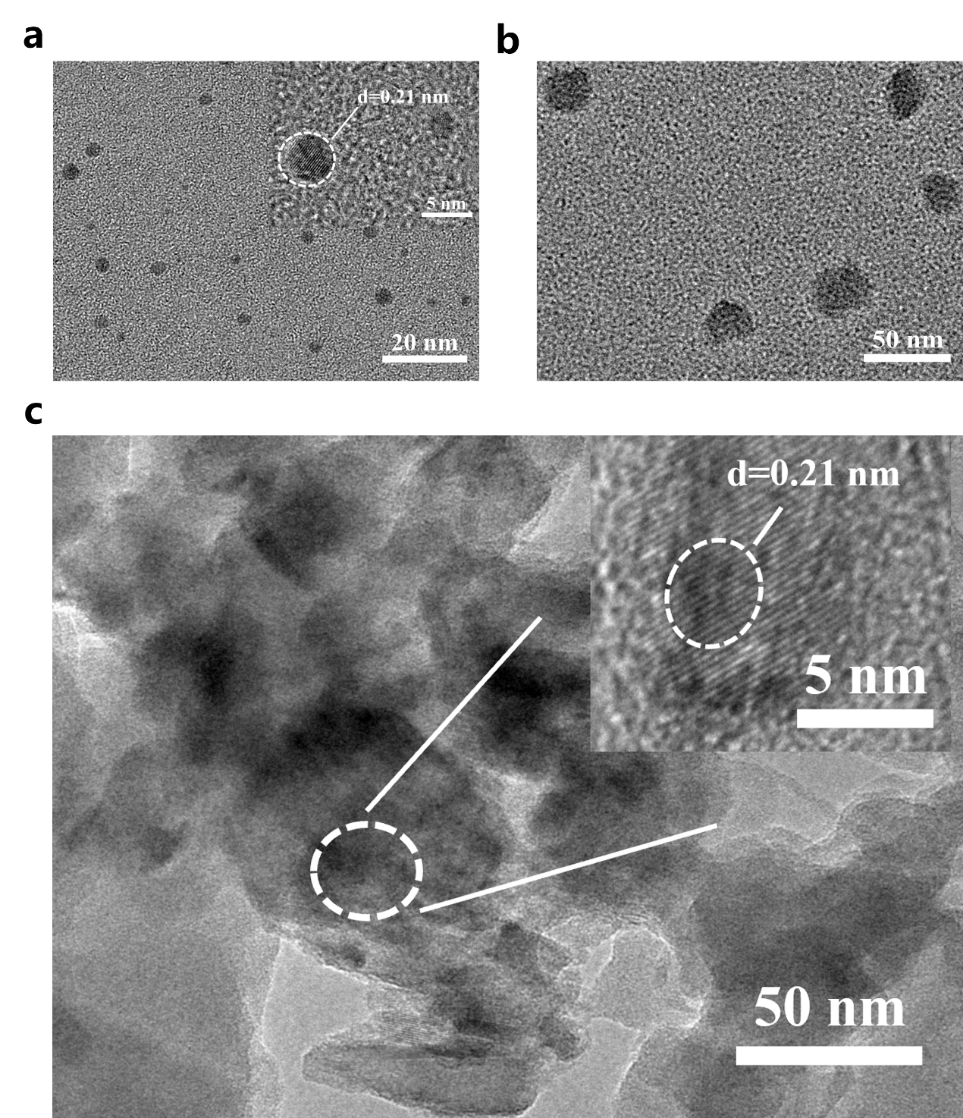
**

**Figure S5.** TEM images of (a) F-CDs (inset: HRTEM image of F-CDs), (b) PA-CDs and (c) HA-CDs (inset: HRTEM image of HA-CDs).

**8. Raman spectrum of HA-CDs**





**Figure S6.** Raman spectrum of HA-CDs.

**9.** **XPS spectra of PA-CDs and HA-CDs**


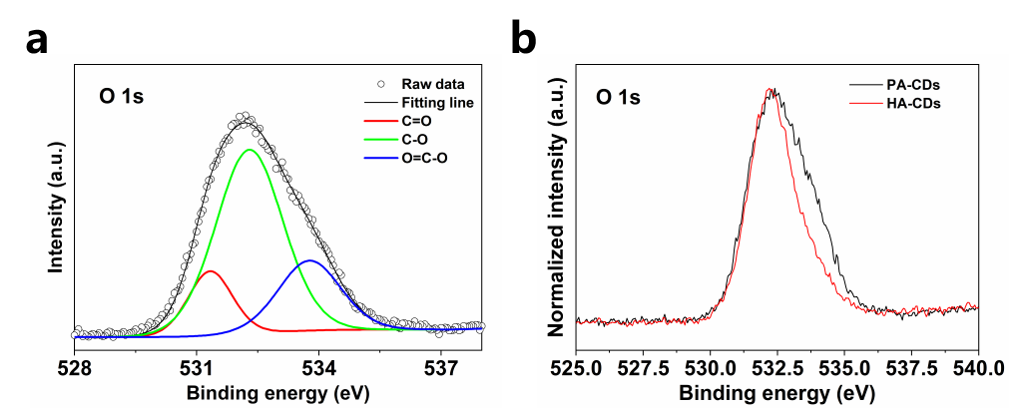


**Figure S7.** (a) High-resolution O 1s XPS spectra of PA-CDs. (b) Comparison of O 1s XPS spectra between PA-CDs and HA-CDs.

**10. FT-IR spectra of PA-CDs and HA-CDs**


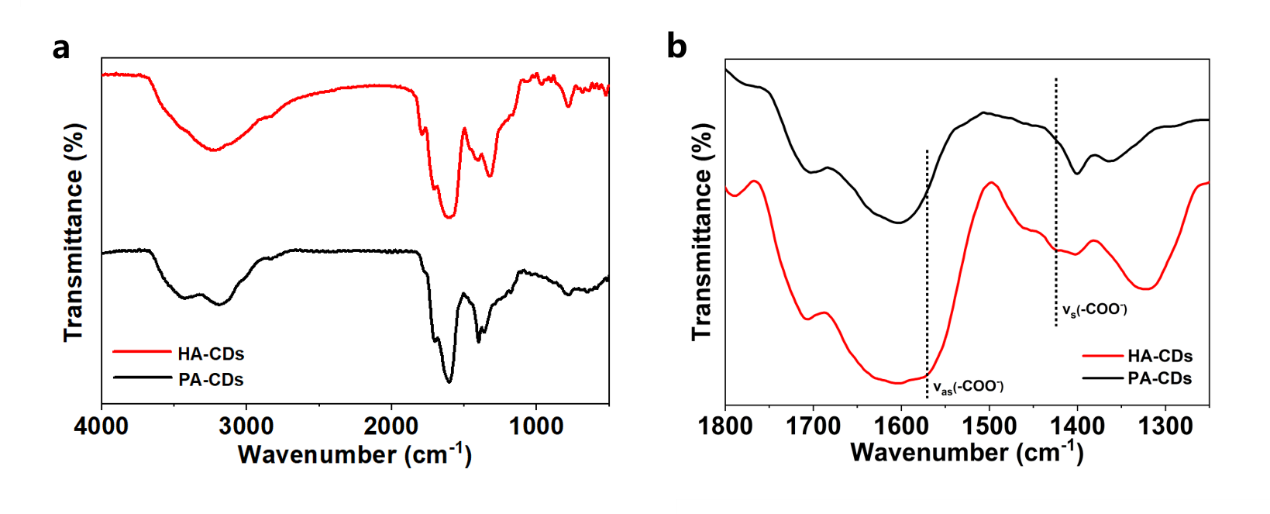


**Figure S8.** Localized magnified FT-IR spectra of PA-CDs and HA-CDs at 1200−1800 cm^-1^.

**11. Zeta potentials of PA-CDs and HA-CDs**

**
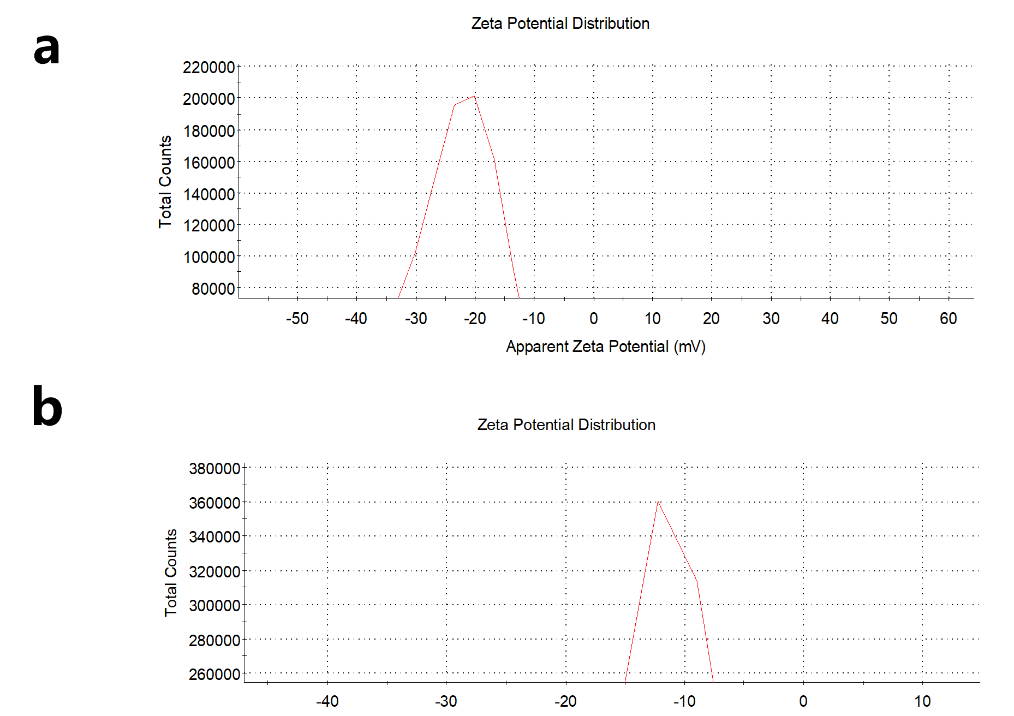
**

**Figure S9.** Zeta potentials of (a) PA-CDs and (b) HA-CDs.

**12.** **Absorption of PA-CDs and HA-CDs in solids**

**
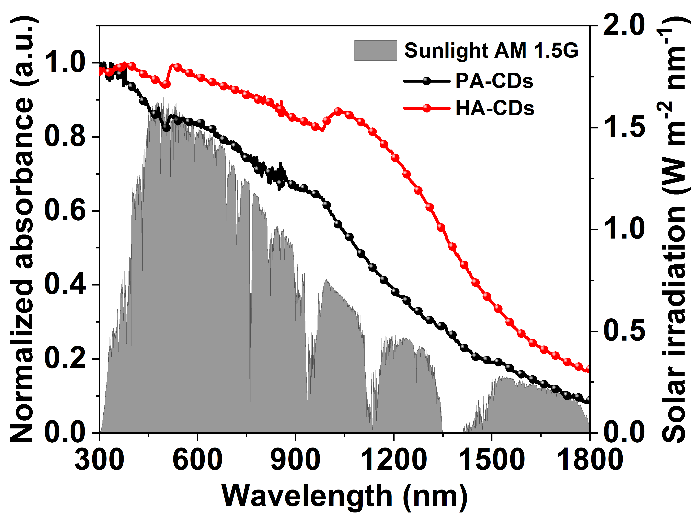
**

**Figure S10.** Absorption spectra of HA-CD and PA-CD powders, alongside the solar radiation spectrum.

**13. Water resistance of PA-CDs and HA-CDs**

**
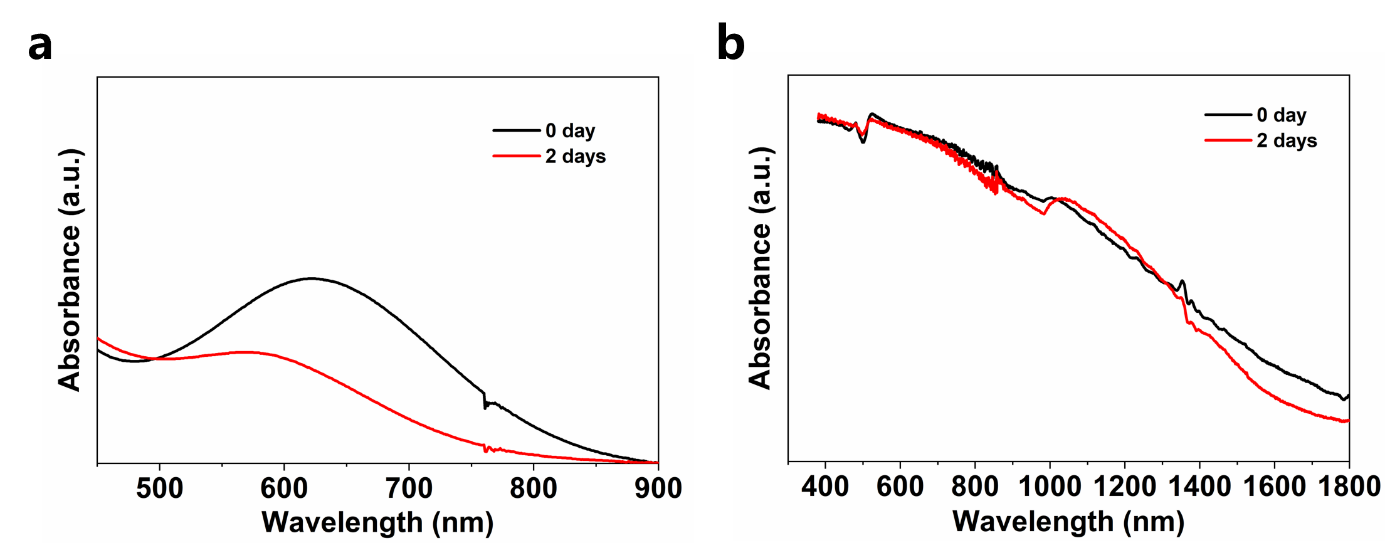
**

**Figure S11.** Absorption spectra of (a) PA-CDs and (b) HA-CDs before and after immersed in H_2_O for 2 days.

**14. Stability of HA-CDs**


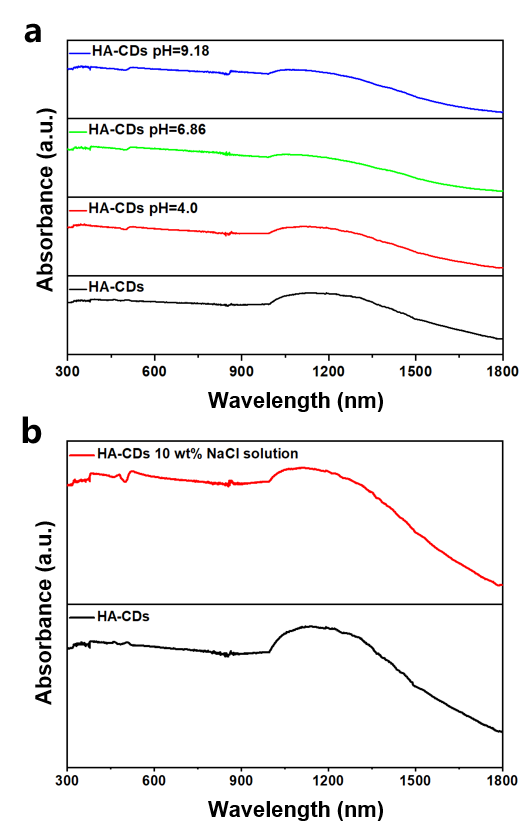


**Figure S12.** (a) The absorption spectra of HA-CDs before and after immersion in aqueous solutions with different pH values (pH = 4.0, 6.86, 9.18) for 12 hours. (b) The absorption spectra of HA-CDs before and after immersion in 10 wt% NaCl aqueous solution.

**15. Photothermal properties of HA-CDs under 1 kW m^-2^ solar irradiation**


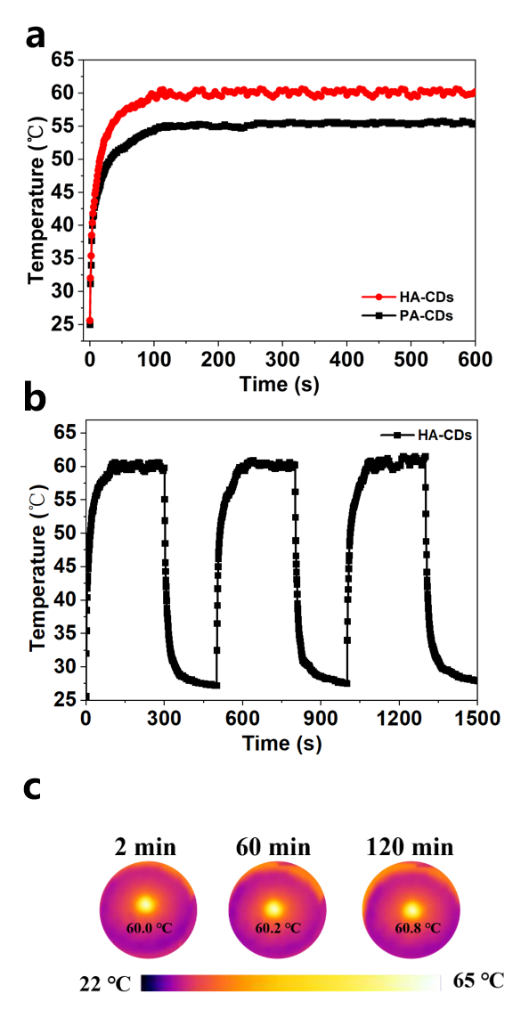


**Figure S13.** (a) Temperature of PA-CDs and HA-CDs as a function of time of under 1 kW m^-2^ solar irradiation. (b) Temperature of HA-CDs as a function of time during 3 cycles of heating-cooling (sunlight on and off) processes. (c) IR thermal images and temperatures of HA-CDs at different time points under continuously irradiation of 1 kW m^-2^ simulated solar irradiation.

**16. Photothermal conversion properties of HA-CDs under irradiation of 1064 nm laser**
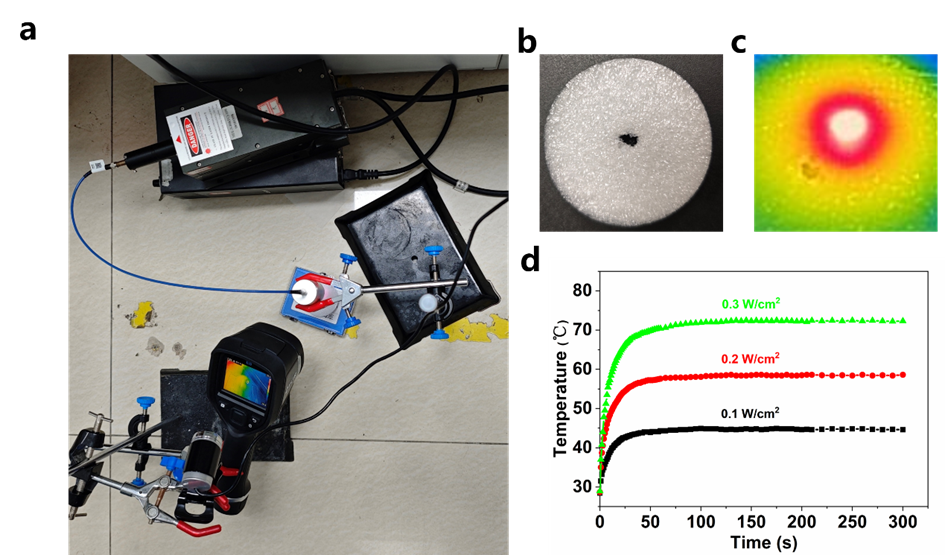


**Figure S14.** (a) Photograph of experiment setup. (b) Digital and (c) IR photos of HA-CDs under irradiation of 1064 nm laser. (d) Temperature of HA-CDs as a function of time under irradiation of 1064 nm laser with different power densities.

**17.** **Water wetting capability of HA-CDs**


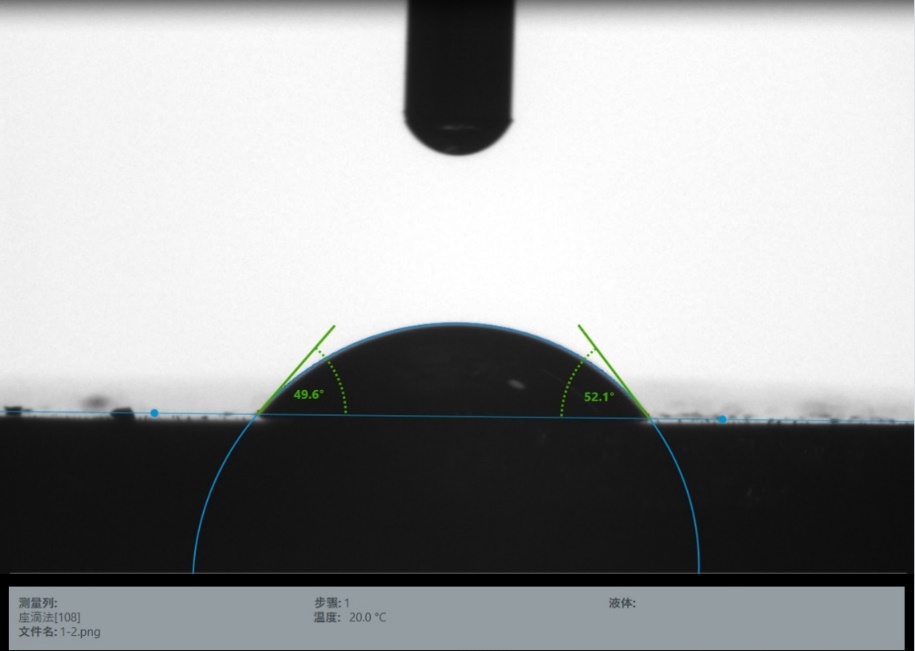


**Figure S15.** Contact angle of water on the surface of HA-CDs.

**18. BET analysis of HA-CDs**


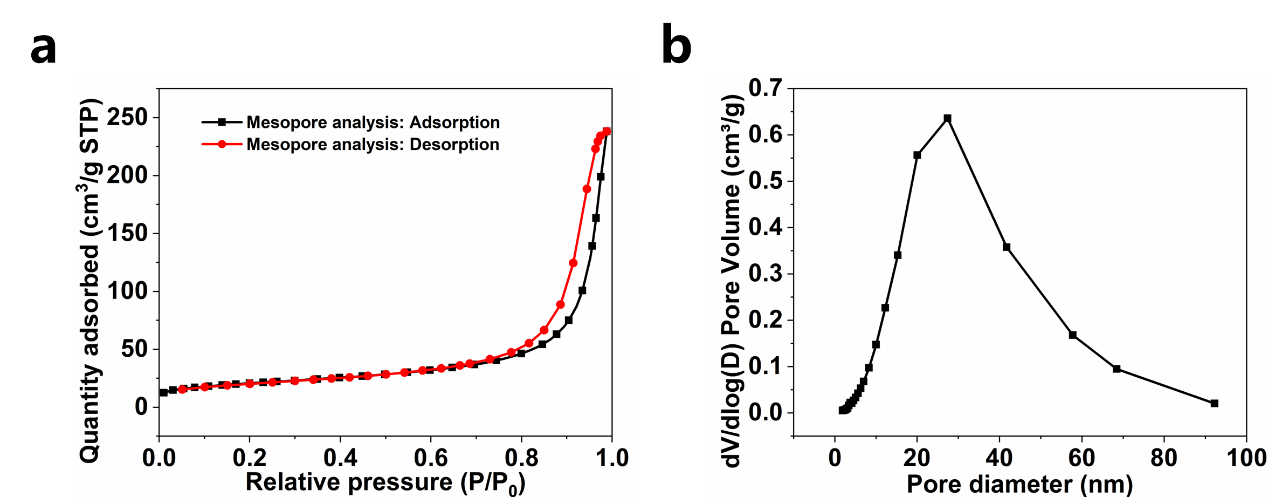


**Figure S16.** (a) Isothermal adsorption-desorption curves of HA-CDs. (b) Pore diameter distribution curve of HA-CDs.

| Sample name | HA-CDs |
| --- | --- |
| BET surface area/m^2^ g^-1^ | 71.6131 |
| Single point adsorption total pore volume of pores/cm^3^ g^-1^ less than 163.3112 nm diameter | 0.368442 |
| t-Plot micropore volume/cm^3^ g^-1^ | 0.005175 |
| BJH Adsorption cumulative volume of pores/cm^3^ g^-1^ between 1.7000 nm and 300.0000 nm diameter | 0.361413 |
| BJH Desorption cumulative volume of pores/cm^3^ g^-1^ between 1.7000 nm and 300.0000 nm diameter | 0.368092 |
| BJH Adsorption average pore diameter/nm | 21.7095 |
| BJH Desorption average pore diameter/nm | 19.0734 |

**Table S4.** N_2_ physisorption analysis using BET model for HA-CDs.

**19. Microstructure of pristine non-woven fabric**





**Figure S17.** SEM image of pristine non-woven fabric.

**20. Water wetting capability of pristine non-woven fabric and HA-CDs-fabric**


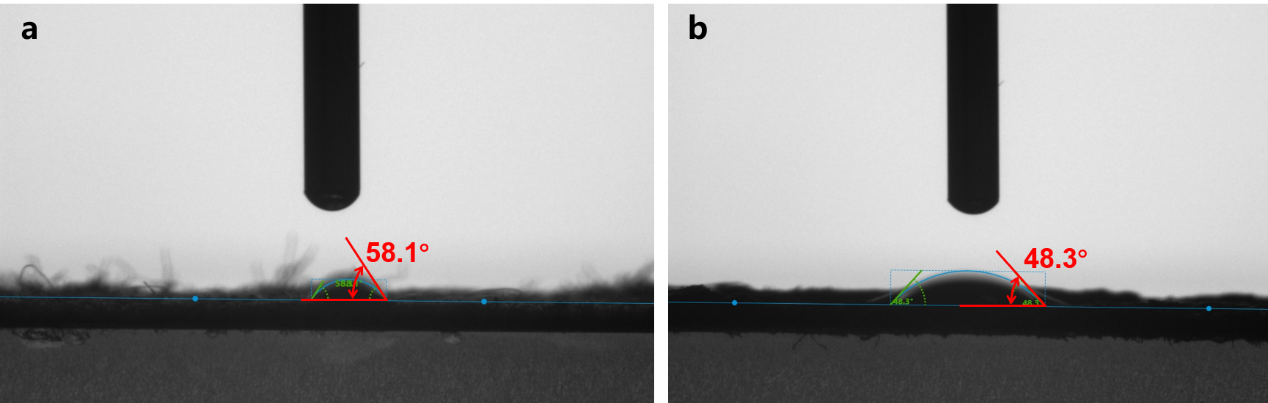


**Figure S18.** Contact angles of water on the surface of (a) pristine non-woven fabric and (b) HA-CDs-fabric.

**21. Water resistance evaluation for PA-CDs-fabric and HA-CDs-fabric**


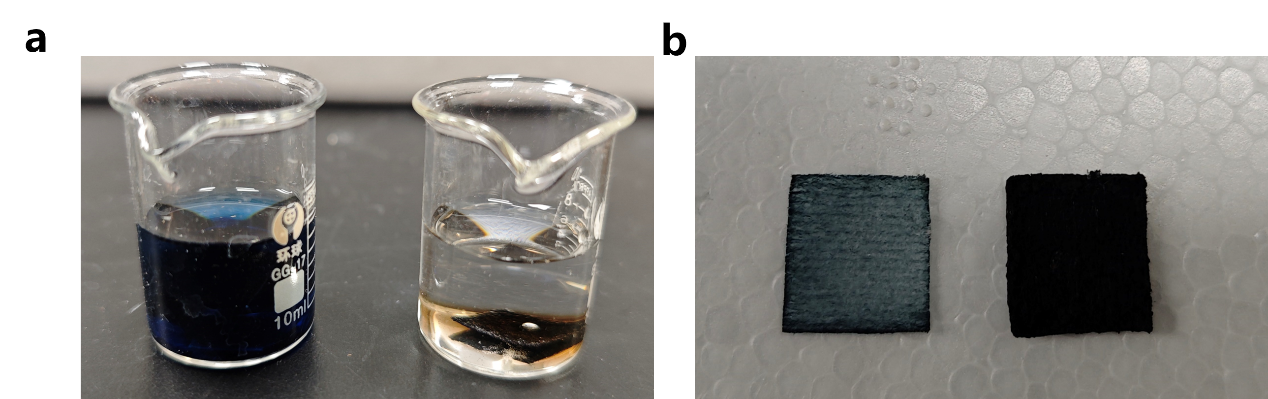


**Figure S19.** Digital photos of (a) PA-CDs-fabric (left) and HA-CDs-fabric (right) immersed in water; (b) PA-CDs-fabric (left) and HA-CDs-fabric (right) removed from water and dried.

**22. Experimental platform of interfacial water evaporation system employing pristine non-woven fabric as the evaporator**


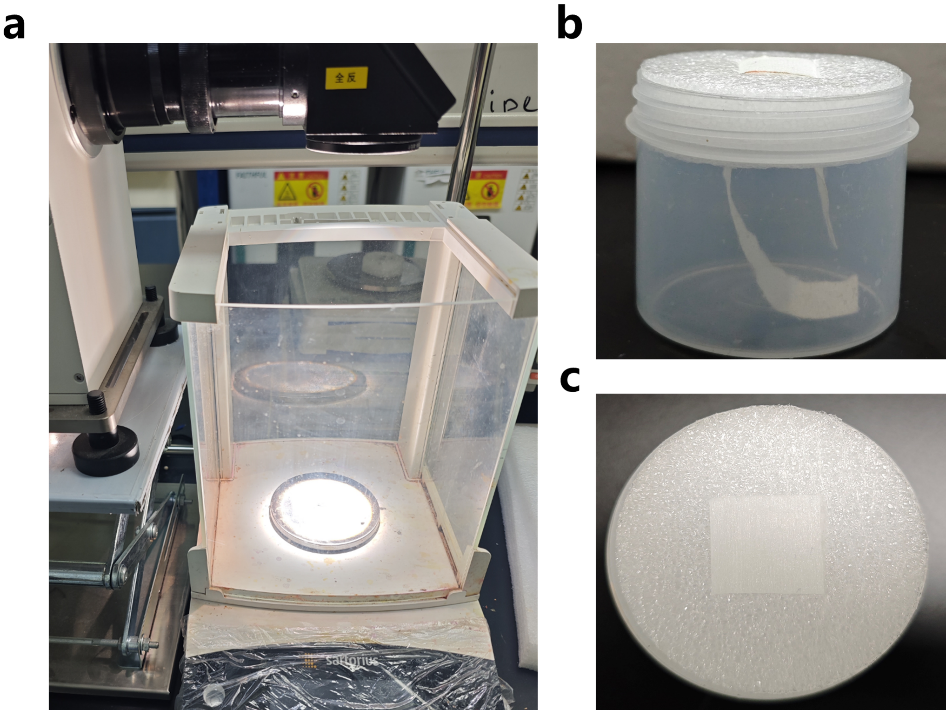


**Figure S20.** (a) Photograph of experiment setup of interfacial water evaporation system. Digital photos of (b, c) 2D (pristine non-woven fabric) evaporation device.

**23. Water evaporation performance of PA-CDs-fabric evaporator**


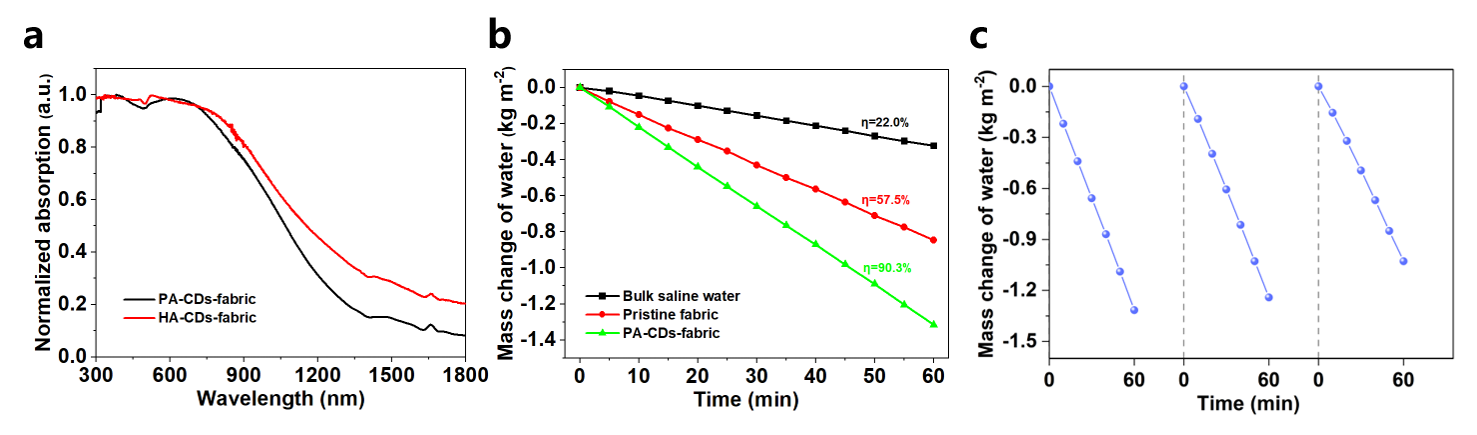


**Figure S21.** (a) Absorption spectra of PA-CDs-fabric and HA-CDs-fabric. (b) Mass loss curves for bulk saline water, evaporation systems with pristine fabric evaporatior for saline water, and with PA-CDs-fabric evaporator for saline water during 1 h of simulated 1 kW m^-2^ solar irradiation. (c) Mass loss curves of PA-CDs-fabric evaporator for three cycles of water evaporation experiments, and the evaporation rates were determined to be 1.32 kg m^-2^ h^-1^, 1.24 kg m^-2^ h^-1^, and 1.03 kg m^-2^ h^-1^ for the 1st, 2nd, 3rd round of evaporation experiment (each cycle of evaporation experiment was conducted for 1 h under simulated 1 kW m^-2^ solar irradiation).

**24.** **Illustration of salt precipitation of the evaporation system employing HA-CDs-fabric evaporator**

**
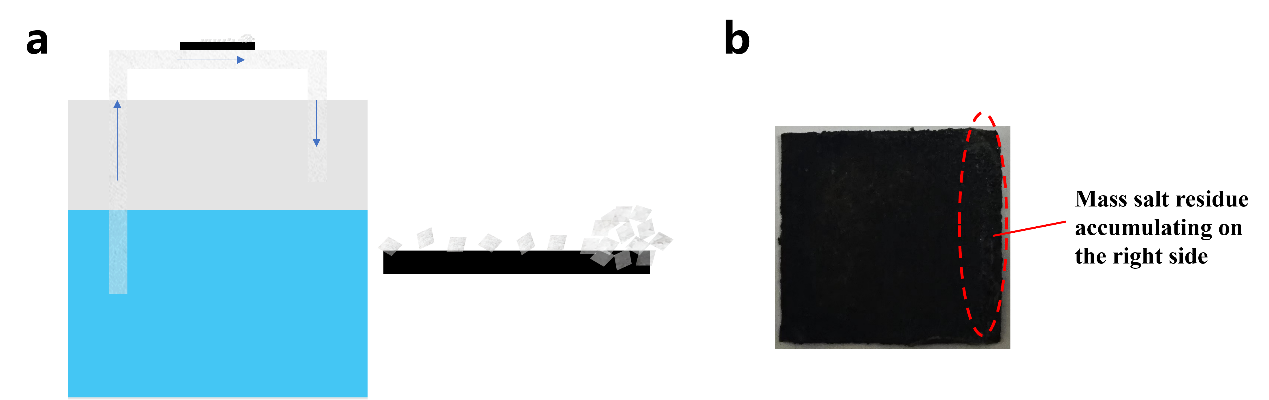
**

**Figure S22.** (a) Schematic diagram of salt precipitation. (b) Photo of HA-CDs-fabric after three rounds of evaporation test.

**25. Salts removal from the surface of the evaporator by water rinsing**

**
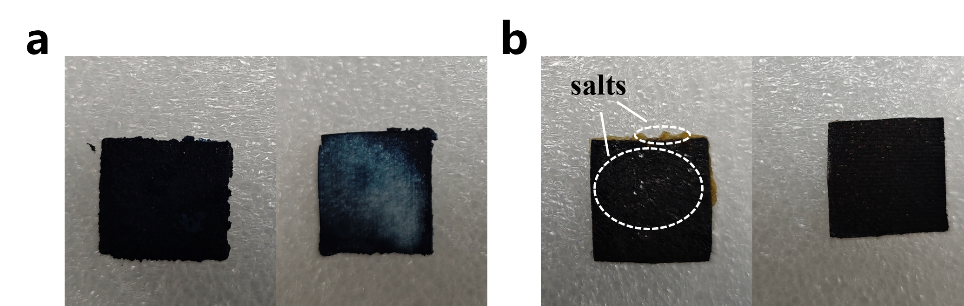
**

**Figure S23.** (a) Photos of PA-CDs-fabric before (left) and after (right) desalination by water rinsing. (b) Photos of HA-CDs-fabric before (left) and after (right) desalination by water rinsing. (Prior to the experiments, salts were sprinkled onto the surfaces of PA-CDs-fabric and HA-CDs-fabric.)

**26. Experimental platform for thermoelectric generation**

The design of the thermoelectric device is illustrated in **Figure** **S24**. The hot side of the TEG is covered by a photothermal material, while the cold side is placed on a circulating water condenser. The output voltage and current were measured using VICTOR 8145C digital multimeter (**Figure 5b** and **Figure S25**). Under solar irradiation, as the photothermal material heats up, a temperature difference is created between the two sides of the TEG, which induces an electrical voltage.

We conducted tests on the output voltage of the TEG under three different conditions: (1) with the hot side directly irradiated by simulated sunlight, (2) with the hot side covered by a pristine non-woven fabric, and (3) with the hot side covered by HA-CDs-fabric. The first two conditions served as references to evaluate the net voltage increase specifically attributed to the photothermal effect of the HA-CDs-fabric.


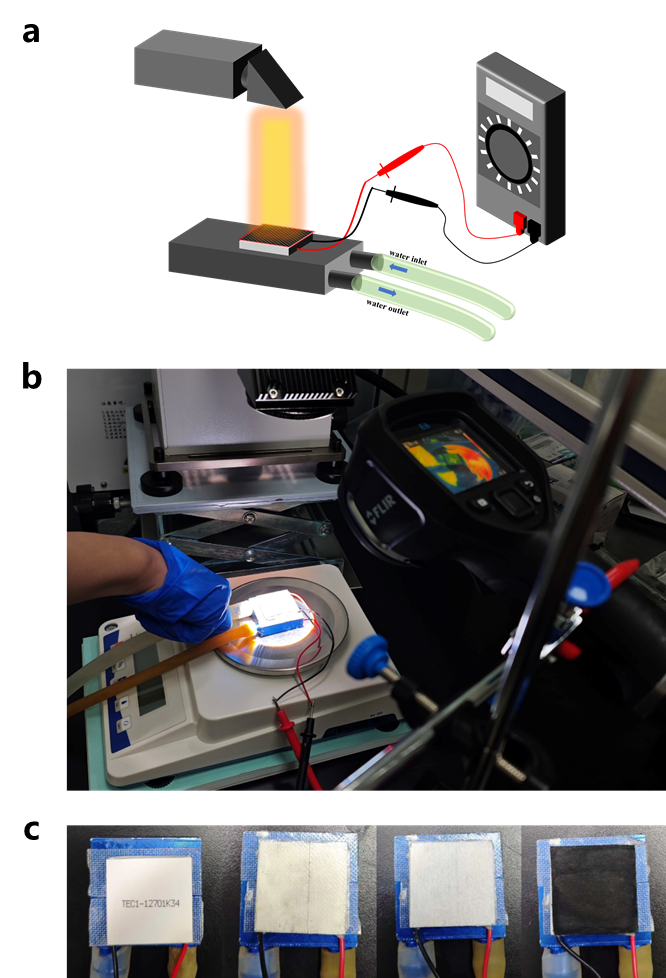


**Figure S24.** (a) Experimental setup diagram of the TEG. (b) Photograph of the experimental setup for thermoelectric generation. (c) Photos of the TEGs with different layers as hot side (from left to right: bare TEG, TEG covered by adhesive, pristine fabric and HA-CDs-fabric, respectively).





**Figure S25.** The voltage and current values generated by the TEG with hot side covered by HA-CD-fabric under 1 kW m^-2^ and 2 kW m^-2^ solar irradiation.

**References**

[1] Y. Wang, Y. Ji, Y. Yang, Z. Chen, H. Sun, X. Wang, Z. Zou, H. Huang, *ACS Energy Lett.* **2024**, *9*, 336.

[2] J. Liu, Y. Cui, Y. Pan, Z. Chen, T. Jia, C. Li, Y. Wang, *Angew. Chem. Int. Ed.* **2022**, *61*, e202117087.

[3] X. Li, G. Ni, T. Cooper, N. Xu, J. Li, L. Zhou, X. Hu, B. Zhu, P. Yao, J. Zhu, *Joule* **2019**, *3*, 1795.
